# ﻿New species and new records of families, genera and species of land snails (Mollusca, Gastropoda) from French Guiana

**DOI:** 10.3897/zookeys.1230.133585

**Published:** 2025-03-06

**Authors:** Olivier Gargominy, Benoît Fontaine, Sandrine Tercerie, Dario Zuccon

**Affiliations:** 1 PatriNat (OFB-MNHN-CNRS-IRD), Muséum national d’Histoire naturelle - CP41, 36 rue Geoffroy Saint-Hilaire 75005 Paris, France; 2 PatriNat (OFB-MNHN-CNRS-IRD), CESCO (MNHN-CNRS-SU), Muséum national d’Histoire naturelle - 3 allée des crapauds - Bat 135 - 43, rue Buffon 75005 Paris, France; 3 Institut de Systématique, Évolution, Biodiversité (ISYEB), Muséum national d’Histoire naturelle, CNRS, Sorbonne Université, EPHE, Université des Antilles, CP 50, 57 rue Cuvier, 75005 Paris, France

**Keywords:** Hidden diversity, Mitaraka, Nouragues, rainforest, Saül, Trinité

## Abstract

This paper presents an investigation of material collected during four intensive collecting trips of land snails in French Guiana from 1995 to 2020 and deposited in the French National Museum of Natural History collections. This material forms the basis of the following novelties: four families are reported for the first time from French Guiana: Diplommatinidae, Cystopeltidae, Thysanophoridae and Strobilopsidae; three native species: *Lyroconusplagioptycha* (Helicoidea, Thysanophoridae), *Pupisomamacneilli* (Pupilloidea, Valloniidae) and *Strobilopsmorsei* (Pupilloidea, Strobilopsidae) and one introduced species *Diplosolenodesoccidentalis* (Veronicelloidea, Veronicellidae) are reported for the first time from French Guiana; five new species are described: *Adelopomaquasimodo* Gargominy, **sp. nov.** (Cyclophoroidea, Diplommatinidae), *Lilloiconchagalbao* Gargominy, **sp. nov.** (Punctoidea, Cystopeltidae), *Protoglyptusbernicolae* Gargominy, **sp. nov.** (Orthalicoidea, Bulimulidae), *Pseudosubulinasanti* Gargominy, **sp. nov.** (Testacelloidea, Spiraxidae), and *Happiadecaensi* Gargominy, **sp. nov.** (Scolodontoidea, Scolodontidae). Finally, *Drymaeussurinamensis* Vernhout, 1914, **syn. nov.** is considered as a new synonym of *Mesembrinuslusorius* (L. Pfeiffer, 1855), and *Drymaeusarcuatostriatus* (L. Pfeiffer, 1855) is proposed as the new identification of *Drymaeusmeesi* sensu Tillier, 1980 non Breure, 1976.

## Abstract (fr)

Cet article présente une étude du matériel collecté lors de quatre missions de collecte intensive d’escargots terrestres en Guyane française de 1995 à 2020, dont le matériel est déposé dans les collections du Muséum national d’Histoire naturelle. Ce matériel a permis de révéler les nouveautés suivantes: quatre familles sont signalées pour la première fois de Guyane française: Diplommatinidae, Cystopeltidae, Thysanophoridae et Strobilopsidae ; trois espèces indigènes: *Lyroconusplagioptycha* (Helicoidea, Thysanophoridae), *Pupisomamacneilli* (Pupilloidea, Valloniidae) et *Strobilopsmorsei* (Pupilloidea, Strobilopsidae) et une espèce introduite *Diplosolenodesoccidentalis* (Veronicelloidea, Veronicellidae) sont signalées pour la première fois de Guyane française ; cinq nouvelles espèces sont décrites: *Adelopomaquasimodo* Gargominy, **sp. nov.** (Cyclophoroidea, Diplommatinidae), *Lilloiconchagalbao* Gargominy, **sp. nov.** (Punctoidea, Cystopeltidae), *Protoglyptusbernicolae* Gargominy, **sp. nov.** (Orthalicoidea, Bulimulidae), *Pseudosubulinasanti* Gargominy, **sp. nov.** (Testacelloidea, Spiraxidae) et *Happiadecaensi* Gargominy, **sp. nov.** (Scolodontoidea, Scolodontidae). Enfin, *Drymaeussurinamensis* Vernhout, 1914, **syn. nov.** est considéré comme un nouveau synonyme de *Mesembrinuslusorius* (L. Pfeiffer, 1855), et *Drymaeusarcuatostriatus* (L. Pfeiffer, 1855) est proposé comme une nouvelle identification de *Drymaeusmeesi* sensu Tillier, 1980 non Breure, 1976.

## ﻿Introduction

French Guiana remains a real field of adventure and discovery for malacologists: access to sites is generally very difficult, land snails are very rare, and very few people make field collections. The very low visible abundance of land snails in the Guianan forest, already observed by early collectors (Drouët 1859), has been confirmed: one can spend whole days in the forest without seeing a single living snail. In a comprehensive revision published in 1980 (Tillier 1980), only 52 species were recorded from the territory of French Guiana. However, large tracts of the forest interior had never been surveyed, and very few minute species were documented, suggesting that the inventory was far from complete.

In this context, in order to fill the gaps in the knowledge of this fauna, surveys (years 1997, 1999, 2015, 2018, 2019, 2020) have been conducted in remote areas of French Guiana in order to inventory the molluscan diversity. They included massive sieving of leaf-litter and revealed a neglected small to minute mollusc fauna (e.g., Gargominy and Ripken 1998; Gargominy and Fontaine 2015; Gargominy et al. 2022). The purpose of this article is to present some of the results of these surveys, including revision of the taxonomy of species collected, except for the Subulininae and the micro Scolodontidae, both of which need a specific paper. Four families previously unknown from French Guiana are documented and five species new to science are described. Material for molecular studies and COI sequences are provided for the first time from the area, although molecular studies are not possible due to lack of comparative material.

Finally, we discuss the reasons this fauna is so poorly known, including the lack of prospecting in French Guiana and neighbouring countries, the rarity of snails, and their patchy distribution in a seemingly homogeneous rainforest.

## ﻿Material and methods

### ﻿Studied material

**Figure 1.**
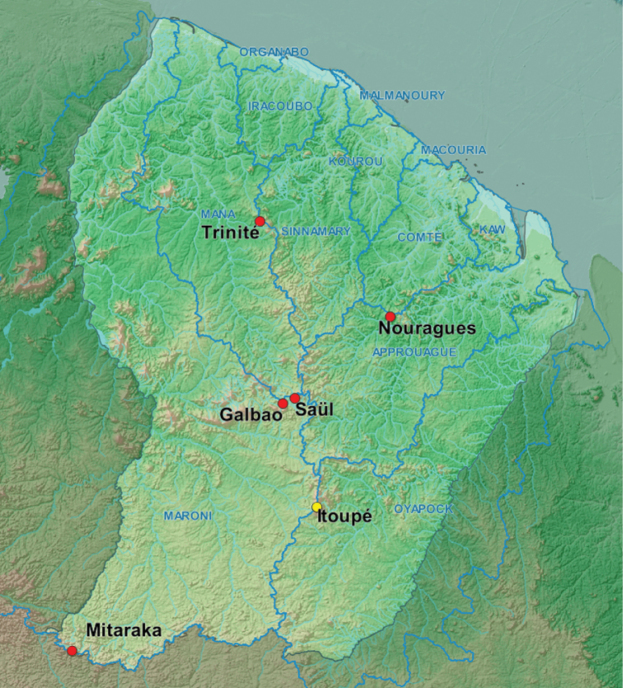
Location of the four main collecting events providing material for this study (Nouragues, Mitaraka, Saül/Galbao, and Trinité, red dots). Itoupé is the type locality for *Happiadecaensi* sp. nov. (yellow dot). Main river basins are represented.

Studied material comes from four main collecting events (Fig. 1):

1. Survey at the Réserve Naturelle Nationale des Nouragues organized by the Muséum national d’Histoire naturelle (**MNHN**) and the Centre National de la Recherche Scientifique (**CNRS**). The scientific station (ca 130 m a.s.l.) is situated at the foot of an inselberg in the Balenfois Mountains (482 m) on the watershed dividing Comté and Approuagues catchments. Molluscs were sampled between 1 and 20 November 1997 (GBIF dataset) and in June 1999 (GBIF dataset) by the first author (OG) and Theo Ripken. Some material has already been the subject of publications and descriptions of new species (Muratov and Gargominy 2011; Gargominy and Muratov 2012).
2. ‘Our Planet Reviewed’ Mitaraka 2015 Survey (GBIF dataset), co-organized by the MNHN and the NGO Pro-Natura International (**PNI**) (Touroult et al. 2018). The expedition was conducted in the Mitaraka Mountains, a largely unknown and uninhabited area in the south-westernmost corner of French Guiana, directly bordering Suriname and Brazil. It forms part of the Maroni river catchment and of the Tumuc-Humac mountain chain, extending east in the Amapá region (Brazil) and west in southern Suriname. Molluscs were sampled between 1 and 24 March 2015 by the first (OG) and second (BF) authors (see report Gargominy and Fontaine 2015).
3. Atlas de la Biodiversité Communale (ABC) of Saül, initiated in 2018 and organized by the Parc Amazonien de Guyane (PAG) with financial support from the Office Français de la Biodiversité (OFB). Saül is a small village situated in the middle of French Guiana at an altitude of 200 m a.s.l. from where Mont Galbao (717 m) can be reached in a day’s walk. Saül and Mont Galbao are situated on the watershed between the Mana and Maroni river catchments. Molluscs were sampled between 16 and 29 November 2018 by the three first authors and Sébastien Sant (PAG), and between 14 and 26 February 2020 by the first (OG) and second (BF) authors, Sébastien Sant (PAG), and Ahmed Abdou (MNHN) (GBIF dataset). The first results were published in Gargominy et al. (2022).
4. Survey at the Réserve naturelle nationale de La Trinité organized by the Office national des Forêts (ONF). This reserve comprises an isolated mountain range with inselbergs (478 m) on the watershed dividing the Maroni and Sinnamary catchments. Molluscs were sampled between 8 and 18 April 2019 by the second (BF) and third (ST) authors (GBIF dataset).

Additional material comes from donations, in particular Thibaud Decaëns and Sébastien Cally from Itoupé (DIADEMA project) on the watershed dividing the Maroni and Camopi/Oyapock catchments.

All the studied material is deposited in the Mollusc collection of the MNHN. Each lot can be traced with its inventory number, MNHN-IM-20XX-XXXX, in the collection database of the MNHN at the following address: https://science.mnhn.fr/institution/mnhn/collection/im/item/search?lang=en_US.

### ﻿Sampling methods

Extensive sieving of leaf litter was conducted in all four field trips. This technique is the only way to approach species inventory completeness, because many species are either small or rare, and many are both. Leaf litter is processed at the collection site using a Winkler sieve (1 cm mesh), with the coarse material checked for snails (empty shells and live animals) and discarded. The remaining material is processed as quickly as possible at the base camp to collect live animals: it is passed through 5 mm, 2 mm, and 0.6 mm sieves. The two larger fractions are carefully examined with the naked eye and the third is sorted under a dissecting microscope. Material passing through the 0.6 mm sieve is discarded. Live molluscs are drowned overnight or prepared with the niku-nuki method (Fukuda et al. 2008) mainly for the smaller ones and fixed in 96% ethanol.

### ﻿DNA barcoding

Total genomic DNA was extracted using the Macherey-Nagel NucleoSpin 96 Tissue Kit and following the manufacturer’s protocol, in combination with the epMotion 5075 robot (Eppendorf). We amplified the 658 bp barcode portion of the mitochondrial cytochrome oxidase I (COI) with primers LCO1490 and HCO2198 (Folmer et al. 1994). Newly obtained sequences were deposited in GenBank and BOLD (Barcode of Life Datasystem).

### ﻿Abbreviations

**AA** Ahmed Abdou

**BF** Benoît Fontaine

**FMNH** Field Museum of Natural History, Chicago, USA

**MNHN** Muséum national d’Histoire naturelle, Paris, France

**NHMUK** Natural History Museum, London, United Kingdom

**OG** Olivier Gargominy

**ONF** Office national des Forêts

**PAG** Parc amazonien de Guyane

**RN** Réserve Naturelle

**ST** Sandrine Tercerie

**TR** Theo E. J. Ripken

## ﻿Systematics

### ﻿Class Gastropoda Cuvier, 1795

**Subclass Caenogastropoda Cox, 1960**

**Order Architaenioglossa Haller, 1890**

**Superfamily Cyclophoroidea Gray, 1847**

**Family Neocyclotidae Kobelt & Möllendorff, 1897**

**Subfamily Amphicyclotinae Kobelt & Möllendorff, 1897**

**Genus *Cyclopedus* Gargominy & Muratov, 2012**

#### ﻿ Cyclopedus anselini

Taxon classificationAnimaliaArchitaenioglossaNeocyclotidae

Gargominy & Muratov, 2012

50C1AF2B-C2CC-521B-AB65-73FB9F4AD749

Fig. 2

Cyclopedus anselini Gargominy & Muratov, 2012: 785, fig. 2.

##### Link.

https://molluscabase.org/aphia.php?p=taxdetails&id=1477355.

##### Type locality.

French Guiana, Régina, Réserve naturelle des Nouragues, Montagnes Balenfois, field station.

##### Type material.

***Holotype*.** French Guiana • 1 dry specimen; Régina, RN des Nouragues, carré N10 du km^2^ layonné (N10); 4.08845°N, 52.67269°W; alt. 140 m; 11 Nov. 1997; TR, OG leg.; leaf litter on lateritic soil in a natural forest gap; MNHN-IM-2000-25066. ***Paratypes*** (3). French Guiana • 1 dry specimen; same data as the holotype; MNHN-IM-2000-25067 • 1 dry specimen; Régina, RN des Nouragues, carré M12 du km^2^ layonné (N7); 4.08699°N, 52.67381°W; alt. 140 m; 08 Nov. 1997; TR, OG leg.; forêt primaire, grand plateau; MNHN-IM-2000-25138 • 1 dry specimen; Régina, RN des Nouragues, carré N15 du km^2^ layonné (N14); 4.08372°N, 52.67503°W; alt. 155 m; 15 Nov. 1997; TR, OG leg.; forêt primaire, pied d’arbre à contrefort (code N-15-138); MNHN-IM-2000-25139.

##### Other material examined.

French Guiana • 42 dry specimens; Régina, RN des Nouragues, carré M12 du km^2^ layonné (N7); 4.08699°N, 52.67381°W; alt. 140 m; 08 Nov. 1997; TR, OG leg.; forêt primaire, grand plateau; MNHN-IM-2018-14184• 2 dry specimens; Régina, RN des Nouragues, carré N10 du km^2^ layonné (N10); 4.08845°N, 52.67269°W; alt. 140 m; 11 Nov. 1997; TR, OG leg.; forêt primaire, chablis; MNHN-IM-2018-14183 • 2 dry specimens; Régina, RN des Nouragues, carré N13 du km^2^ layonné (NB5); 4.08603°N, 52.67353°W; alt. 170 m; 03 Jun. 1999-29 Jun. 1999; TR, OG leg.; MNHN-IM-2018-14292 • 4 95% ethanol specimen; Saül, Mont Galbao (SAUL47); 3.60183°N, 53.27239°W; alt. 650 m; 27 Nov. 2018; OG, SS, ST, BF (PAG & MNHN) leg.; DZ brulée et cambrouse à l’est; GenBank: PQ629106; Bold: DREAL124-23; MNHN-IM-2013-75859; GenBank: PQ629100; Bold: DREAL123-23; MNHN-IM-2013-75860; GenBank: PQ629099; Bold: DREAL122-23; MNHN-IM-2013-75861; GenBank: PQ629094; Bold: DREAL121-23; MNHN-IM-2013-75862 • 2 dry specimens; Saül, same data as preceding; MNHN-IM-2012-21975 • 3 dry specimens; Saül, Cascades du Mont Galbao, face nord-est (SAUL64); 3.60376°N, 53.26121°W; alt. 320 m; 19 Feb. 2020; BF, AA, OG (PAG & MNHN) leg.; Bordure de cambrouse; MNHN-IM-2018-14064 • 2 95% ethanol specimens; Saül, Mont Galbao face nord-est (SAUL65); 3.59681°N, 53.26257°W; alt. 503 m; 20 Feb. 2020; BF, AA, OG (PAG & MNHN) leg.; Cambrouse et lianes en forêt; MNHN-IM-2013-76396 • 2 dry specimens; Saül, Mont Galbao face nord-est (SAUL66); 3.59995°N, 53.26697°W; alt. 382 m; 20 Feb. 2020; BF, AA, OG (PAG & MNHN) leg.; MNHN-IM-2018-14139 • 2 dry specimens; Saül, Mont Galbao face nord-est (SAUL68); 3.60018°N, 53.25877°W; alt. 251 m; 21 Feb. 2020; BF, AA, OG (PAG & MNHN) leg.; Bord de cambrouse, sous lianes; MNHN-IM-2018-14029 • 2 dry specimens; Mana, Réserve de la Trinité, layon inselberg 120m (pente inselberg) (TRI21); 4.61157°N, 53.40833°W; alt. 180 m; 15 Apr. 2019; BF, ST (MNHN/ONF) leg.; pied de rochers granitiques; MNHN-IM-2014-7310.

##### Remarks.

Mont Galbao and Montagnes de la Trinité represent two new records for this species previously only known from its type locality in Nouragues (Gargominy and Muratov 2012), suggesting a broader distribution probably extending outside French Guiana.

**Figure 2.**
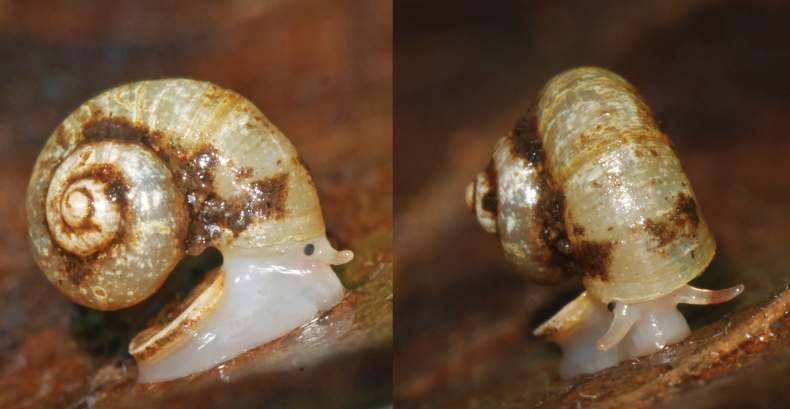
*Cyclopedusanselini* from Mont Galbao (MNHN-IM-2013-75862).

The first living specimens are reported here. The body is totally white but the area around and below the eyes is lightly pink; two quite distinct black eyes at the outer base of the tentacles; tentacle slightly conical, with the outer third pale pink (Fig. 2). From the four sequenced specimens (all from the same locality) we recovered two haplotypes differing by one synonymous mutation only.

### ﻿Family Diplommatinidae L. Pfeiffer, 1847

**Genus *Adelopoma* Doering, 1885**

#### ﻿ Adelopoma quasimodo

Taxon classificationAnimaliaArchitaenioglossaNeocyclotidae

Gargominy sp. nov.

4C0AA735-850B-5857-848F-6324AA1CC5F2

https://zoobank.org/5EDCF9CB-1E0B-46BB-8983-577469F12790

Figs 3 , 4 , 5

##### Type locality.

French Guiana, Saül, northeastern foothills of Bœuf Mort mountain.

##### Type material.

***Holotype*.** French Guiana • 1 dry specimen; Saül, Versant nord de Bœuf Mort, le long du sentier de Grand Bœuf Mort (SAUL44); 3.64098°N, 53.21863°W; alt. 300 m; 24 Nov. 2018; OG, SS, ST, BF (PAG & MNHN) leg.; Pied d’arbre à contreforts; MNHN-IM-2012-21233. ***Paratypes*** (7). French Guiana • 1 95% ethanol specimen; same data as the holotype; GenBank: PQ629098; Bold: DREAL176-23; MNHN-IM-2013-75603 • 1 95% ethanol specimen; Saül, Versant nord-est de Bœuf Mort, le long du sentier de Grand Bœuf Mort (SAUL37); 3.63555°N, 53.21035°W; alt. 340 m; 21 Nov. 2018; OG, SS, ST, BF (PAG & MNHN) leg.; Autour d’un arbre à contreforts; GenBank: PQ629107; Bold: DREAL373-23; MNHN-IM-2013-75648 • 1 95% ethanol specimen and 4 dry specimens; same data as preceding; MNHN-IM-2013-75652 and MNHN-IM-2012-21955 respectively.

##### Other material examined.

French Guiana • 9 dry specimens; Saül, Cascades du Mont Galbao, face nord-est (SAUL64); 3.60376°N, 53.26121°W; alt. 320 m; 19 Feb. 2020; BF, AA, OG (PAG & MNHN) leg.; Bordure de cambrouse; MNHN-IM-2018-14065 • 13 dry specimens; Saül, Mont Galbao face nord-est (SAUL65); 3.59681°N, 53.26257°W; alt. 503 m; 20 Feb. 2020; BF, AA, OG (PAG & MNHN) leg.; Cambrouse et lianes en forêt; MNHN-IM-2018-14047 • 1 95% ethanol specimen; same data as preceding; MNHN-IM-2013-76395 • 1 dry specimen; Saül, Mont Galbao face nord-est (SAUL66); 3.59995°N, 53.26697°W; alt. 382 m; 20 Feb. 2020; BF, AA, OG (PAG & MNHN) leg.; MNHN-IM-2018-14143 • 1 95% ethanol specimen; same data as preceding; MNHN-IM-2013-76473 • 5 dry specimens; Saül, Mont Galbao face nord-est (SAUL68); 3.60018°N, 53.25877°W; alt. 251 m; 21 Feb. 2020; BF, AA, OG (PAG & MNHN) leg.; Bord de cambrouse, sous lianes; MNHN-IM-2018-14032 • 1 95% ethanol specimen; same data as preceding; MNHN-IM-2013-76384.

##### Diagnosis.

An *Adelopoma* species of minute size, more ovate than conical, densely ribbed, and with a deep suture.

##### Description.

Holotype: Shell minute (height 2.0 mm, diameter 1.1 mm), sinistral, thin, elongate, conical in first whorls then cylindrical; colour white, subtranslucent. Whorls 5.2, inflated, strongly rounded, separated by a deep suture. Protoconch 1.5 whorls, smooth; protoconch/teleoconch transition distinct because of change in sculpture; surface of teleoconch with lamellate axial ribs (27 on body whorl) and distinct minute spiral striae between the ribs. Body whorl rounded, ventrolaterally with a distinct bulge through which an inner, palatal, crescent-shaped denticle can be seen. Aperture almost circular; upper insertion of the peristome not descending; peristome continuous but the upper part largely merged to the preceding whorl, double, weakly expanded and thickened; there is a columellar lamella at the bottom of the columella, which can be fully seen only by looking through the aperture (also indistinctly visible in frontal view in the aperture); perforate, umbilicus marked by the continuation of the axial ribs. Operculum unknown.

**Figure 3.**
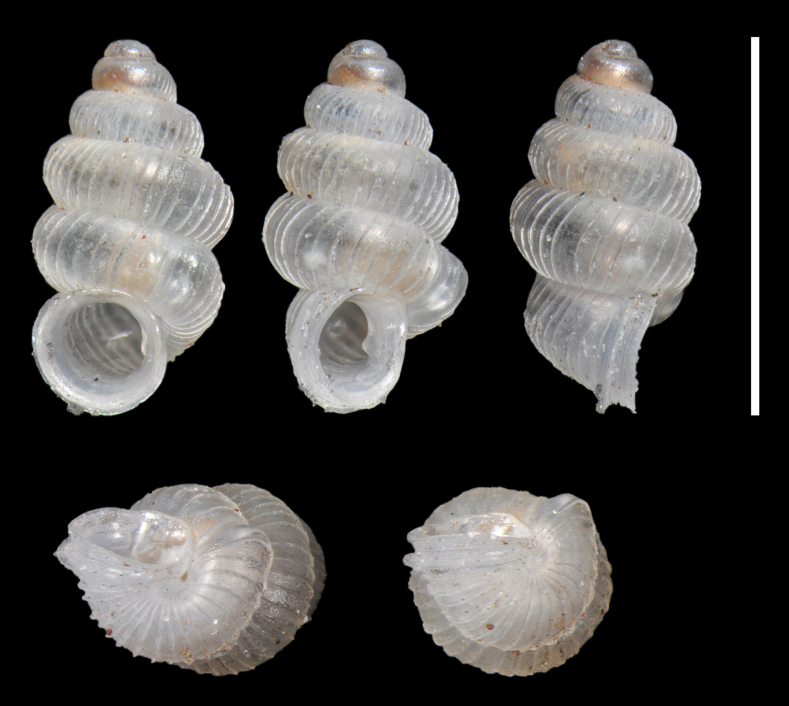
*Adelopomaquasimodo* sp. nov., holotype MNHN-IM-2012-21233. Scale bar: 2 mm.

Paratypes: Operculum ~ 0.5 mm, corneous, concave, almost completely circular in shape, completely transparent and whitish. Body (5) colourless and transparent; except for the ca two first whorls where the light brown odd caesura can be seen by transparency. Eyes black, quite minute, at the base of the tentacles. Tentacles ~ 0.2 mm long, cylindrical, extending upward.

**Figure 4.**
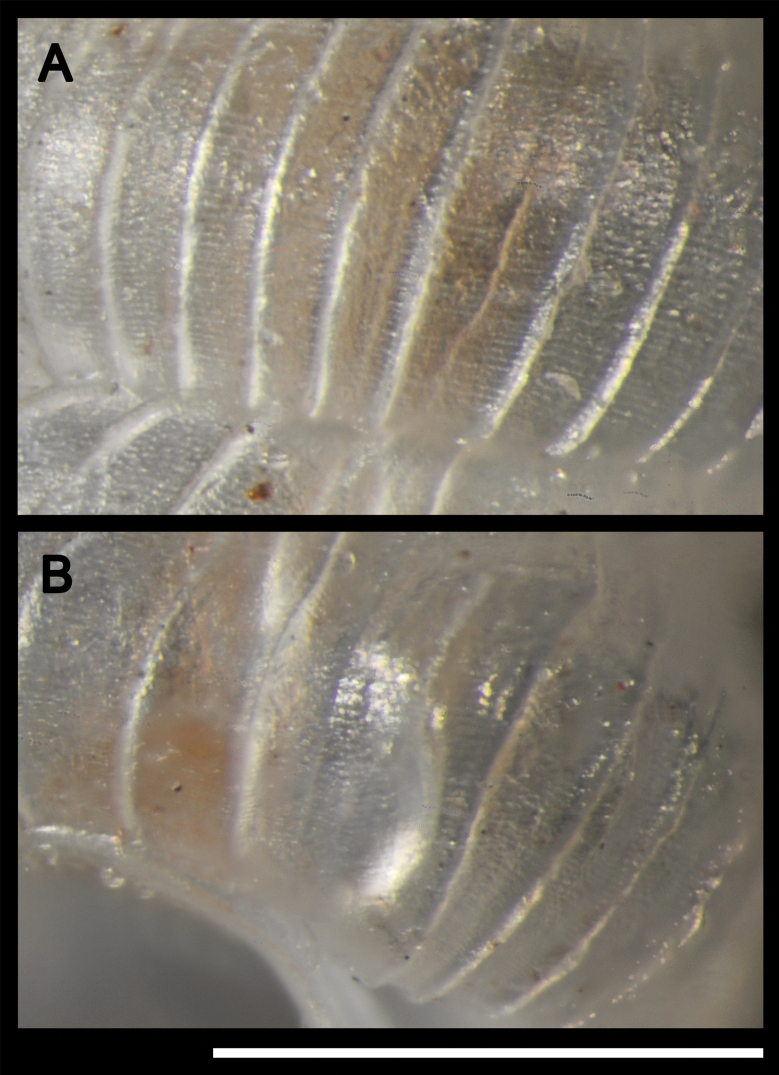
*Adelopomaquasimodo* sp. nov., holotype MNHN-IM-2012-21233: **A** detail of antepenultimate whorl showing spiral microsculpture **B** detail of the bulge of body whorl showing denticle inside the shell by transparency. Scale bar: 0.5 mm.

##### Etymology.

The species is named after Quasimodo, the hunchback character of Victor Hugo’s novel Notre-Dame de Paris, and refers to the bulge on the body whorl. It also reminds one of the catastrophic event of the fire at Notre-Dame de Paris cathedral on 15 April 2019, which occurred during the Trinité collecting trip. Treated as a noun in apposition.

**Figure 5.**
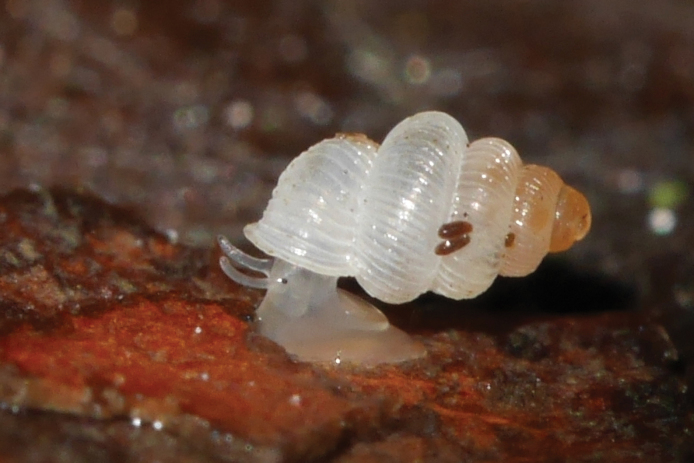
*Adelopomaquasimodo* sp. nov., paratype MNHN-IM-2013-75652 crawling on a dead leaf with two faeces on its shell.

##### Distribution.

This species is only known from Saül, on the northeastern foothills of both Bœuf Mort and Galbao mountains.

##### Habitat.

Primary forest, under leaf litter on alkaline soils.

##### Remarks.

This species shares with *Adelopomaperuvianum* Hausdorf & Munoz, 2004 the distinct bulge on the body whorl, the presence of a columellar lamella and the distinct perforation which are distinctive characteristics of *A.peruvianum* according to the original description (Hausdorf and Munoz 2004). It differs from that species, together with *Adelopomabrasiliense* Morretes, 1954 (unfortunately not mentioned in Hausdorf and Munoz (2004) but the holotype was illustrated by Simone (2006: 46) later) in being smaller, more cylindrical and less conical, the sutures deeper, and with a higher rib density. Hausdorf and Munoz (2004) mentioned that the columellar denticle has been overlooked in other species; the presence of the distinct bulge on the body whorl might also have been ignored because it is hardly visible even if fully developed as in *A.quasimodo*. Thus the whole genus would need a revision over its large distribution.

We obtained two COI sequences differing by nine mutations. Both are related to one sequence of *Adelopomatucma* (Döring, 1884) from Argentina, type species of the genus (*p*-distance 6.1–6.4%, GenBank HM753341; Webster et al. 2012).

The genus *Adelopoma* is known from Mexico to Peru, northern Argentina (Tucumán), south-eastern Brazil, Venezuela, and Trinidad (Torre et al. 1942; Hausdorf and Munoz 2004; Martins and Simone 2014). Its occurrence in French Guiana is not surprising, but its scarcity is remarkable as it is only known from foothills around Saül, on slopes oriented east-northeast on alkaline complexes. However, it is probable that it has been overlooked and that its range in French Guiana is larger than the Saül area.

### ﻿Subclass Heterobranchia Burmeister, 1837

**Infraclass Euthyneura**

**Subterclass Tectipleura Schrödl, Jörger, Klussmann- Kolb & N. G. Wilson, 2011**

**Superorder Eupulmonata Haszprunar & Huber, 1990**

**Order Stylommatophora A. Schmidt, 1855**

**Suborder Helicina Rafinesque, 1815**

**Superfamily Punctoidea Morse, 1864**

**Family Cystopeltidae Cockerell, 1891**

**Genus *Lilloiconcha* Weyrauch, 1965**

#### ﻿ Lilloiconcha galbao

Taxon classificationAnimaliaArchitaenioglossaNeocyclotidae

Gargominy sp. nov.

31E03958-D2D2-5780-929D-768B7EF0014B

https://zoobank.org/CA9FA418-C36E-4AF2-92B5-01A754807284

Figs 6 , 7

##### Type locality.

French Guiana, Saül, Mont Galbao.

##### Type material.

***Holotype*.** French Guiana • 1 dried specimen; Saül, Mont Galbao (SAUL47); 3.60183°N, 53.27239°W; alt. 650 m; 27 Nov. 2018; OG, SS, ST, BF (PAG & MNHN) leg.; DZ brulée et cambrouse à l’est; MNHN-IM-2013-75668. ***Paratypes*** (20). French Guiana • 1 95% ethanol specimen; same data as the holotype; GenBank: PQ629092; Bold: DREAL909-23; MNHN-IM-2013-75966 • 3 95% ethanol specimens; same data as the holotype; MNHN-IM-2013-75667 • 1 dry specimen; same data as the holotype; MNHN-IM-2012-21986 • 4 dry adult specimens; same data as the holotype; MNHN-IM-2018-896 • 11 dry juvenile specimens; same data as the holotype; MNHN-IM-2018-897.

##### Diagnosis.

A *Lilloiconcha* species with more than five whorls, as high as large, with a small umbilicus and strong ribs.

##### Description.

Holotype: Shell minute (height 2.2 mm, diameter 2.6 mm), dextral, globose, dome-shaped to gibbous, thin; colour corneous; whorls 5.4, inflated, rounded, separated by a deep suture; spire coiling regularly increasing. Protoconch two whorls, smooth; protoconch/teleoconch transition distinct because of change in sculpture; teleoconch sculpture of regular radial ribs, 60 on the body whorl, and a reticulate pattern consisting of fine growth-striae and dense microscopical spiral threads between the ribs. Body whorl rounded. Aperture almost circular; upper insertion of the peristome not descending towards the aperture; suture impressed. Peristome simple, sharp, neither expanded nor thickened. Umbilicus U-shaped, almost cylindrical, contained 3.8× in the shell greater diameter.

**Figure 6.**
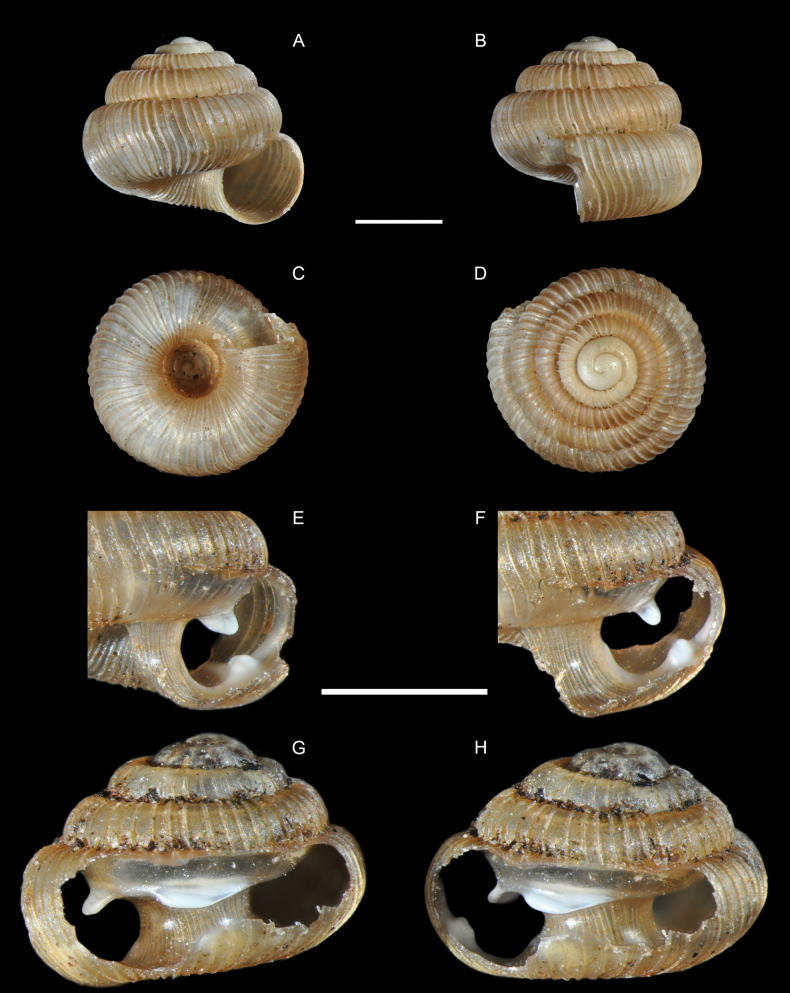
*Lilloiconchagalbao* sp. nov.: **A–D** holotype MNHN-IM-2013-75668**E–H**, paratype MNHN-IM-2012-21986, details of dentition inside the body whorl after the shell has been broken. Scale bars: 1 mm.

**Figure 7.**
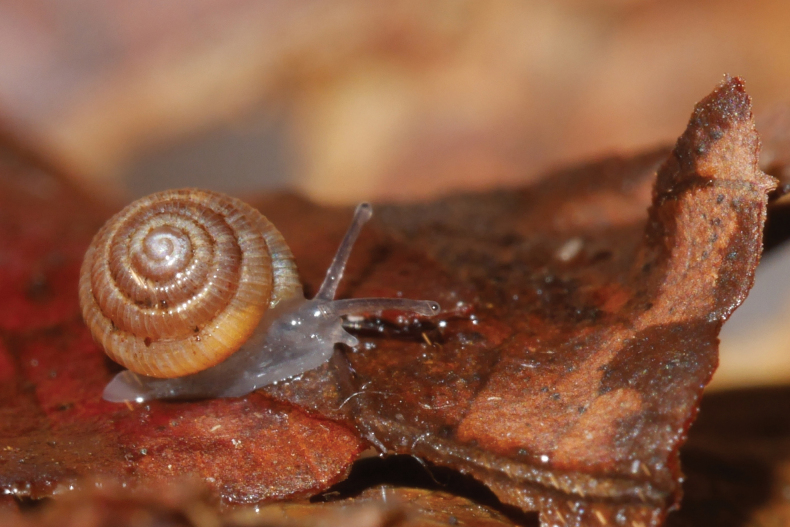
*Lilloiconchagalbao* sp. nov., holotype MNHN-IM-2013-75668 crawling on a dead leaf.

Animal greyish, sole paler; tail without distinct caudal pit, not distinctly truncated; ocular tentacles long and gracile, inflated at the tip; eyes black, small, on the upper front of the tentacles (Fig. 7).

Paratypes: Juveniles have internal lamellae which are dissolved (not present) in adult specimens (more than 4.5–5 whorls, as the holotype): a longitudinal lamella in the middle of the parietal wall formed by up to three (one or two in subadult specimens) elongate, high (~ 40% of the aperture diameter) teeth separated by gaps of half their length, covering ~ 1/8 whorl in length; at the opposite palatal side of each parietal tooth, there is a transversal lamella, extending from the outermost side of the aperture towards half of the columellar wall, principally thickened in front of its corresponding parietal tooth and extending a little bit inside along the whorl. In early juveniles this palatal lamella is reduced to a simple denticle. The lamellar complex reduces the aperture diameter to half its value.

##### Etymology.

The species is named after the type locality, Mont Galbao; treated as a noun in apposition.

##### Distribution.

This species is only known from its type locality, Mont Galbao.

##### Habitat.

Primary forest, under leaf litter on alkaline soils.

##### Remarks.

This species is attributed to genus *Lilloiconcha* Weyrauch, 1965 based on the conchological characters given by Weyrauch (1965), who established the name for a single species which usually has basal and parietal denticles in juvenile stages, *Austrodiscussuperbustucumanus* Hylton Scott, 1963 (see Schileyko 2001; Hausdorf 2005; Miquel et al. 2007). The anatomy of the new species is not known: the only adult specimen is the holotype and its body is dried and contracted inside the shell, thus not available for anatomy without cracking the shell. However, it would not help much for generic attribution as the anatomy of the type species of *Lilloiconcha* and its related genera (for example *Radioconus* Baker, 1927, type species *Helixbactriola* Guppy, 1868) are not known, as indicated by Hausdorf (2005).

We provide the COI sequence of one paratype which relates to the congeneric *Lilloiconchasuperba* from Brazil (*p*-distance 12.5%, GenBank MN792606; Salvador et al. 2020).

The presence of *Lilloiconcha* in French Guiana is not surprising regarding the distribution of the widespread *Lilloiconchagordurasensis* (Thiele, 1927) (Hausdorf 2005). However, the distribution of the genus in French Guiana seems to be limited to the mountainous habitats of the Galbao range, as it has never been collected on the Mitaraka, Trinité, or Nouragues mountains despite intensive collecting. The new species represents the first record of the superfamily Punctoidea and of the family Cystopeltidae in French Guiana.

### ﻿Infraorder Helicoidei

**Superfamily Helicoidea Rafinesque, 1815**

**Family Thysanophoridae Pilsbry, 1926**

**Genus *Lyroconus* H.B. Baker, 1927**

#### ﻿ Lyroconus plagioptycha

Taxon classificationAnimaliaArchitaenioglossaNeocyclotidae

(Shuttleworth, 1854)

4E61151D-D4BA-54B2-B9BF-05D3CE3E9A5D

Figs 8 , 9

Helix plagioptycha Shuttleworth, 1854: 37.
Thysanophora plagioptycha (Shuttleworth, 1854)— Pilsbry (1920): 94, figs 2, 3.
Lyroconus plagioptycha (Shuttleworth, 1854)— Schileyko (2006): 1896.

##### Link.

https://molluscabase.org/aphia.php?p=taxdetails&id=1065439.

##### Material examined.

French Guiana • 1 dry specimen; Régina, Montagne Favard (Montagne de Kaw), ca 200 m sur le chemin W depuis la fin de la route (dégrad) D6 (COT03); 4.5°N, 52.05722°W; 20 Oct. 1997; TR, OG leg.; Petit ruisseau, forêt; MNHN-IM-2012-21187 • 1 dry specimen; Remire-Montjoly, Lotissement DDE, 1.4 km sur la route des plages (D1) après le carrefour de Dégrad des Cannes, Montagne du Mahury (COT06); 4.86056°N, 52.25917°W; alt. 15 m; 21 Oct. 1997; TR, OG leg.; culture de bananes et papayes; MNHN-IM-2012-23924 • 3 dry specimens; Roura, Crique Sourou, Rte forestière de Nancibo (COT10); 4.67273°N, 52.42825°W; alt. 10 m; 23 Oct. 1997; TR, OG leg.; ripisylve, sol alluvial; MNHN-IM-2022-2131 • 1 dry specimen; Saint-Laurent-du-Maroni, Rte du Plateau des cascades, plateau des mines, 3.7 km après la D11 (COT27); 5.38569°N, 54.06283°W; 28 Oct. 1997; TR, OG leg.; reste de forêt sur une route bordée de parcelles conquises sur la forêt; MNHN-IM-2022-2130 • 1 dry specimen; Sinnamary, Petit Saut, 1.4 km avant le rond-point “barrage du Petit Saut” (COT30); 5.06111°N, 52.99778°W; 28 Oct. 1997; TR, OG leg.; forêt dense, fond de talweg; MNHN-IM-2022-2137 • 24 dry specimens; Saül (SAUL25); 3.61274°N, 53.21626°W; alt. 174 m; 17 Nov. 2018; OG, SS, ST, BF (PAG & MNHN) leg.; Pied d’arbre à contrefort; MNHN-IM-2012-21932 • 5 95% ethanol specimens; Saül (SAUL25); 3.61274°N, 53.21626°W; alt. 174 m; 17 Nov. 2018; OG, SS, ST, BF (PAG & MNHN) leg.; Pied d’arbre à contrefort; MNHN-IM-2013-75621 • 2 dry specimens; Saül (SAUL37); 3.63555°N, 53.21035°W; alt. 276 m; 21 Nov. 2018; OG, SS, ST, BF (PAG & MNHN) leg.; Autour d’un arbre à contreforts; MNHN-IM-2012-21956 • 2 95% ethanol specimens; Saül (SAUL37); 3.63555°N, 53.21035°W; alt. 276 m; 21 Nov. 2018; OG, SS, ST, BF (PAG & MNHN) leg.; Autour d’un arbre à contreforts; MNHN-IM-2013-75655 • 4 dry specimens; Saül, Versant nord de Bœuf Mort, le long du sentier de Grand Bœuf Mort (SAUL44); 3.64098°N, 53.21863°W; alt. 300 m; 24 Nov. 2018; OG, SS, ST, BF (PAG & MNHN) leg.; Pied d’arbre à contreforts; MNHN-IM-2012-21965 • 1 95% ethanol specimen; Saül, Cascades au pied du Galbao (SAUL49); 3.60254°N, 53.26051°W; alt. 220 m; 29 Nov. 2018; OG, SS, ST, BF (PAG & MNHN) leg.; Chaos rocheux; GenBank: PQ629103; Bold: DREAL353-23; MNHN-IM-2013-75961 • 1 dry specimen; Saül, Cascades au pied du Galbao (SAUL49); 3.60254°N, 53.26051°W; alt. 220 m; 29 Nov. 2018; OG, SS, ST, BF (PAG & MNHN) leg.; Chaos rocheux; MNHN-IM-2018-677 • 1 95% ethanol specimen; Mana, Layon C vers 1900m (TRI19); 4.583°N, 53.40587°W; alt. 50 m; 14 Apr. 2019; BF, ST (MNHN/ONF) leg.; forêt de lianes; GenBank: PQ629104; Bold: DREAL358-23; MNHN-IM-2013-75993 • 1 95% ethanol specimen; Mana, cambrouse layon C à environ 1300 m du camp Aya (TRI20); 4.58417°N, 53.4054°W; alt. 80 m; 14 Apr. 2019; BF, ST (MNHN/ONF) leg.; Cambrouse; MNHN-IM-2013-75819 • 3 dry specimens; same data as preceding; MNHN-IM-2014-7922 • 2 dry specimens; Mana, Layon inselberg 120m (pente inselberg) (TRI21); 4.61157°N, 53.40833°W; alt. 180 m; 15 Apr. 2019; BF, ST (MNHN/ONF) leg.; pied de rochers granitiques; MNHN-IM-2014-7923 • 1 95% ethanol specimen; Mana, Réserve naturelle de La Trinité, Layon C à environ 1600m du camp Aya (TRI31); 4.58421°N, 53.40542°W; alt. 67 m; 18 Apr. 2019; BF, ST (MNHN/ONF) leg.; Cambrouse; GenBank: PQ629101; Bold: DREAL364-23; MNHN-IM-2013-75992 • 1 95% ethanol specimen; Mana, Réserve naturelle de La Trinité, Layon C à environ 1600m du camp Aya (TRI31); 4.58421°N, 53.40542°W; alt. 67 m; 18 Apr. 2019; BF, ST (MNHN/ONF) leg.; Cambrouse; MNHN-IM-2013-75820 • 12 dry specimens; Mana, Réserve naturelle de La Trinité, Layon C à environ 1600m du camp Aya (TRI31); 4.58421°N, 53.40542°W; alt. 67 m; 18 Apr. 2019; BF, ST (MNHN/ONF) leg.; Cambrouse; MNHN-IM-2014-7924 • 2 dry specimens; Saül, Chemin des Monts la Fumée (SAUL63); 3.63119°N, 53.20586°W; alt. 177 m; 18 Feb. 2020; BF, AA, OG (PAG & MNHN) leg.; Pied d’arbre à contrefort; MNHN-IM-2018-14083 • 1 dry specimen; Saül, Cascades du Mont Galbao, face nord-est (SAUL64); 3.60376°N, 53.26121°W; alt. 320 m; 19 Feb. 2020; BF, AA, OG (PAG & MNHN) leg.; Bordure de cambrouse; MNHN-IM-2018-14067 • 3 dry specimens; Saül, Mont Galbao face nord-est (SAUL65); 3.59681°N, 53.26257°W; alt. 503 m; 20 Feb. 2020; BF, AA, OG (PAG & MNHN) leg.; Cambrouse et lianes en forêt; MNHN-IM-2018-14051 • 1 95% ethanol specimen; Saül, Mont Galbao face nord-est (SAUL65); 3.59681°N, 53.26257°W; alt. 503 m; 20 Feb. 2020; BF, AA, OG (PAG & MNHN) leg.; Cambrouse et lianes en forêt; MNHN-IM-2013-76405 • 1 dry specimen; Saül (SAUL70); 3.60864°N, 53.21492°W; alt. 212 m; 23 Feb. 2020; BF, AA, OG (PAG & MNHN) leg.; MNHN-IM-2018-14120.

**Figure 8.**
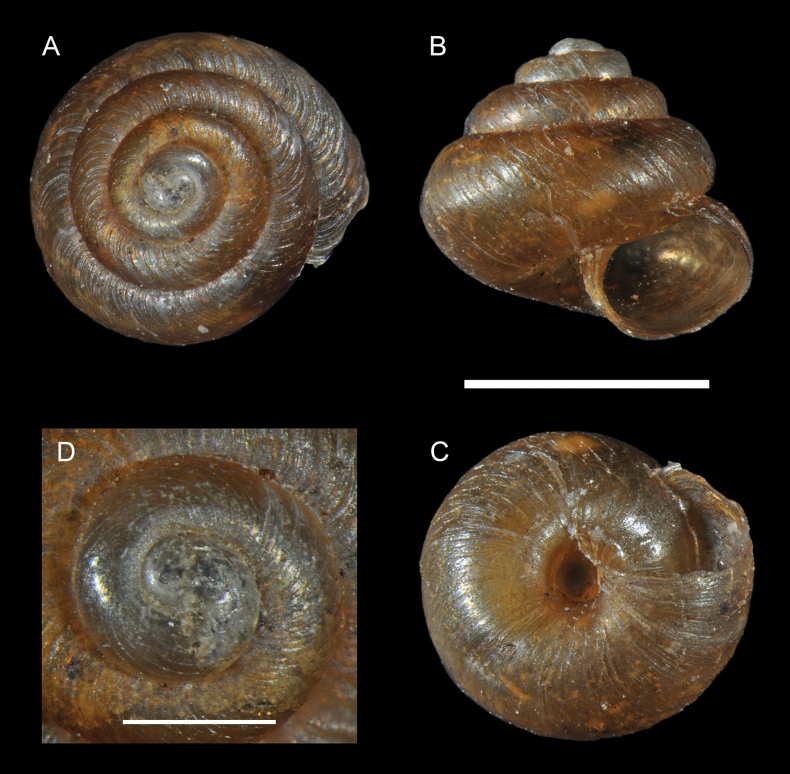
*Lyroconusplagioptycha*, Saül, MNHN-IM-2012-21932. Scale bars: 2 mm (**A–C**); 0.5 mm (**D**).

##### Remarks.

All specimens collected in French Guiana are consistent with the diagnosis by Pilsbry (1920) and the illustration of syntype NMBE 18878 figured in Neubert and Gosteli (2003: pl. 20 fig. 4).

*Lyroconusplagioptycha* (type locality Puerto Rico) is a widespread species in Greater Antilles and Central America from Florida to Venezuela (https://www.gbif.org/species/5190501), and was recently recorded in northeastern Brazil (Salvador et al. 2021). It was reported from Suriname as early as 1960 (Altena 1960). Until now it has never been recorded from French Guiana and this is the first record of the family Thysanophoridae in this territory.

**Figure 9.**
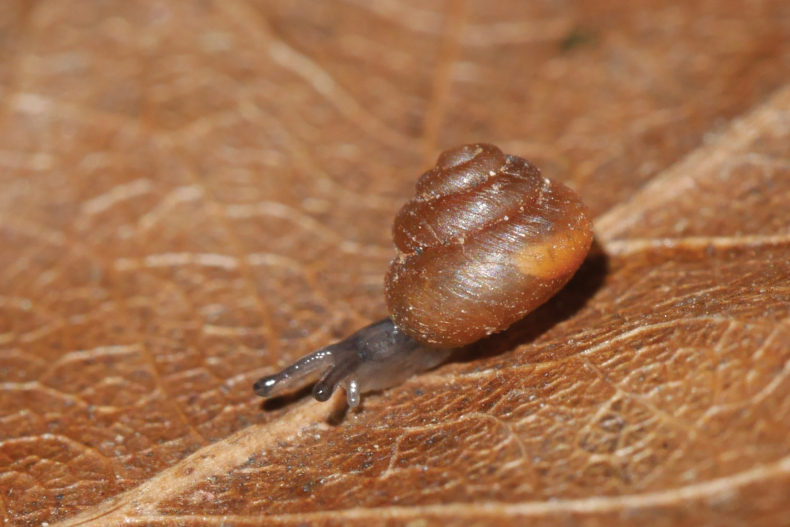
*Lyroconusplagioptycha* from the southern ridge of Bœuf Mort, Saül.

In French Guiana, the species is known from 10 to 500 m a.s.l. both on coastal areas (e.g., Saint-Laurent, Cayenne, Kaw mountain) as well as in the interior in Saül and Trinité.

Two haplotypes were recovered, one from Mana and the other from Saül. Despite their large genetic difference (*p*-distance 2.7%), the specimens appear identical in morphology.

### ﻿Infraorder Orthalicoidei

**Superfamily Orthalicoidea Martens, 1860**

**Family Bulimulidae Tryon, 1867**

**Subfamily Bulimulinae Tryon, 1867**

**Genus *Protoglyptus* Pilsbry, 1897**

#### ﻿ Protoglyptus bernicolae

Taxon classificationAnimaliaArchitaenioglossaNeocyclotidae

Gargominy sp. nov.

A4F3844D-E45A-580A-B93C-EFE947CC541B

https://zoobank.org/4794633F-BE95-4418-9B93-C93A29D6C6FB

Figs 10 , 11

##### Type locality.

French Guiana, Saül, Mont Galbao.

##### Type material.

***Holotype*.** French Guiana • 1 95% ethanol specimen (shell separated); Saül, Mont Galbao (SAUL47); 3.60183°N, 53.27239°W; alt. 650 m; 27 Nov. 2018; OG, SS, ST, BF (PAG & MNHN) leg.; DZ brulée et cambrouse à l’est; GenBank: PQ629105; Bold: DREAL855-23; MNHN-IM-2013-75865. ***Paratypes*** (14). French Guiana • 2 95% ethanol specimens; Saül, Mont Galbao, same data as the holotype; GenBank: PQ629096; Bold: DREAL1376-23; MNHN-IM-2013-75863; GenBank: PQ629093; Bold: DREAL856-23; MNHN-IM-2013-75864 • 1 dry specimen; Saül, Mont Galbao, same data as the holotype; MNHN-IM-2012-21977 • 4 dry specimens; Saül, Mont Galbao face nord-est (SAUL65); 3.59681°N, 53.26257°W; alt. 503 m; 20 Feb. 2020; BF, AA, OG (PAG & MNHN) leg.; Cambrouse et lianes en forêt; MNHN-IM-2018-14041 • 4 95% ethanol specimens; Saül, Mont Galbao, same data as preceding; MNHN-IM-2013-76397• 3 95% ethanol specimen; Mana, Réserve de la Trinité, cambrouse layon C à environ 1300 m du camp Aya (TRI20); 4.58417°N, 53.4054°W; alt. 80 m; 14 Apr. 2019; BF, ST (MNHN/ONF) leg.; Cambrouse; GenBank: PQ629097; Bold: DREAL859-23; MNHN-IM-2013-75989; GenBank: PQ629102; Bold: DREAL858-23; MNHN-IM-2013-75990; GenBank: PQ629111; Bold: DREAL857-23; MNHN-IM-2013-75991.

##### Other material examined.

French Guiana • 4 dry specimens; Mana, Réserve de la Trinité, cambrouse layon C à environ 1300 m du camp Aya (TRI20); 4.58417°N, 53.4054°W; alt. 80 m; 14 Apr. 2019; BF, ST (MNHN/ONF) leg.; Cambrouse; MNHN-IM-2014-7920 • 5 dry juvenile specimens; Mana, Réserve naturelle de La Trinité, Layon C à environ 1600 m du camp Aya (TRI31); 4.58421°N, 53.40542°W; alt. 67 m; 18 Apr. 2019; BF, ST (MNHN/ONF) leg.; Cambrouse; MNHN-IM-2014-7921.

##### Diagnosis.

A *Protoglyptus* species with whorls slightly keeled at the periphery and spiral rows of long setae mainly placed on this keel.

##### Description.

Holotype. Shell of medium size (height 6.0 mm, diameter 5.5 mm), dextral, thin, shiny, medium-spired, conical; colour uniformly brownish, almost subtranslucent. Whorls 4.5, slightly inflated with shallow suture, slightly keeled at the periphery; last whorl descending more rapidly below the periphery of the preceding. Protoconch one whorl, with sculpture consisting of oblique radial ribs, interstices ~ 3× as wide as the ribs, with very fine spiral striae in between the riblets; protoconch/teleoconch transition distinct because of change in sculpture; surface of teleoconch with sculpture consisting, on the upper part of the whorl, of 6–10 fine spiral rows of periostracal scales expanding to long setae at the periphery of the whorl and shorter setae at the middle of its upper part; on the lower part, ~ 16 fine spiral rows of periostracal scales expanding to shorter setae on three rows; this spiral sculpture on the lower part of the whorl totally disappearing from the aperture into the interior of the shell. Peristome not formed, simple; aperture oblique, prosocline, crescent-like. Umbilicus very small, punctiform, partially covered by the columellar wall.

**Figure 10.**
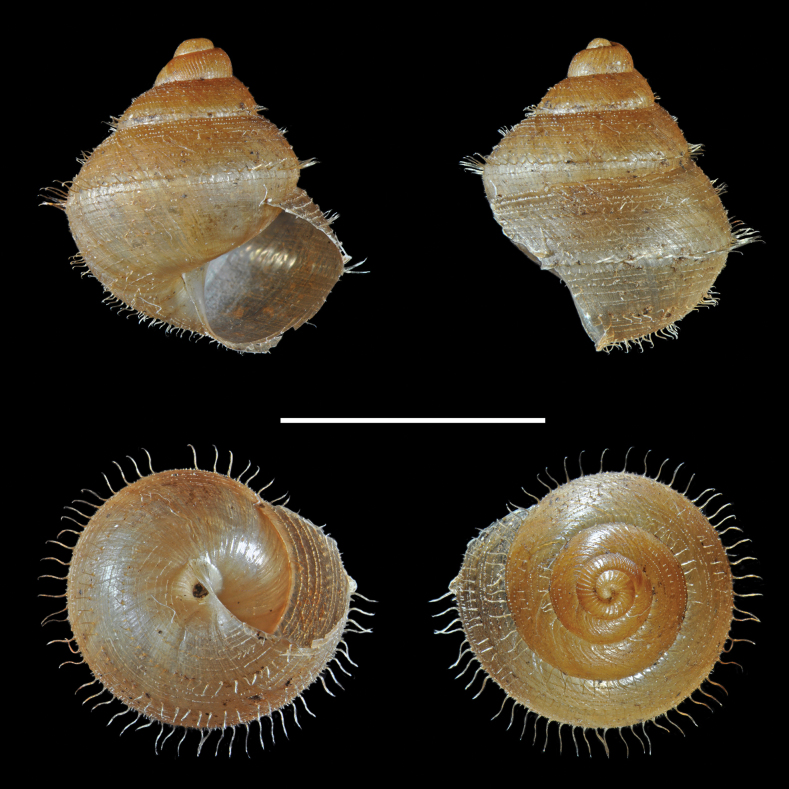
*Protoglyptusbernicolae* sp. nov., holotype MNHN-IM-2013-75865. Scale bar: 5 mm.

**Figure 11.**
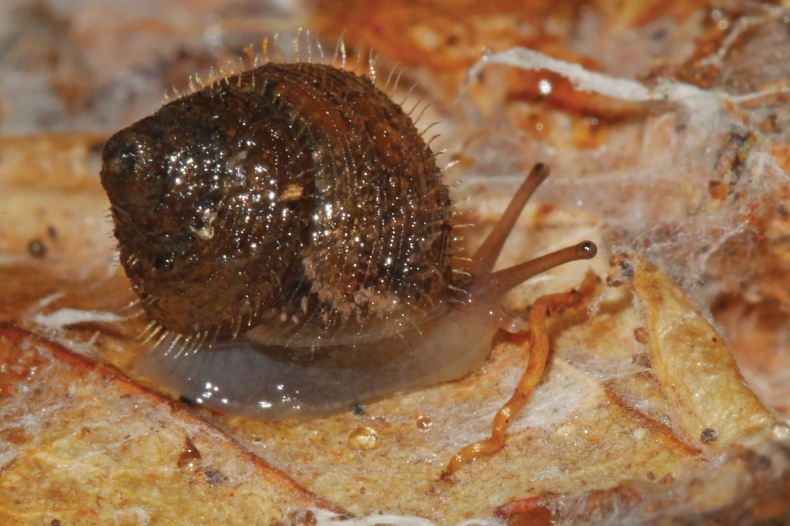
*Protoglyptusbernicolae* sp. nov.: holotype MNHN-IM-2013-75865 alive.

Body pale grey, whitish around the head; head brownish, similar to the colour of the shell; upper tentacles elongated, inflated at the tip; eyes black, small, situated on the upper part of the tentacles; lower tentacles with whitish tips. Inner mantle with small white patches visible through the transparency of the shell (Fig. 11).

##### Etymology.

The species is named after Bernard and Nicole Gargominy, parents of the first author, in recognition of the unswerving taste for nature they have transmitted. The species name is a contraction of both first names declined in the feminine genitive as it ends with Nicole.

##### Distribution.

This species is only known from French Guiana: Mont Galbao and Réserve de la Trinité.

##### Habitat.

In leaf litter of vegetation dominated by *Lasiacis* species (“cambrouses”).

##### Remarks.

No fully adult specimens have been found. The holotype is the more subadult specimen and the aperture description is thus not informative. The structure of the protoconch immediately places the species as belonging to genus *Protoglyptus*. *Protoglyptuslongiseta* (S. Moricand, 1846) described from Bahia Province, Brazil (Moricand 1846: 156, pl. 5 figs 18–20) shares the long setae but is larger, as large as tall, has more convex whorls, and setae of equal length all over the teleoconch.

This species seems to be restricted to vegetation dominated by *Lasiacis* species (Poaceae) locally called “cambrouses”.

The six COI sequences (four haplotypes) form two groups, from Saül and Trinité, respectively, differing by 2.1–2.7% (*p*-distance).

### ﻿Subfamily Peltellinae Gray, 1855

**Genus *Drymaeus* Albers, 1850**

#### ﻿ Drymaeus arcuatostriatus

Taxon classificationAnimaliaArchitaenioglossaNeocyclotidae

(L. Pfeiffer, 1855)

4F2B1AC5-14E5-595E-B9B6-A4DFC4CDC5CB

Figs 12 , 13

Bulimus arcuatostriatus L. Pfeiffer, 1855: 95: ”Peru” ”Long. 30, lat. 13 mill.” (Cuming coll.).
Drymaeus (Drymaeus) arcuatostriatus (L. Pfeiffer, 1855)—Breure and Ablett 2014: 23, fig. 41A–C (lectotype NHMUK1975455, dimensions H 27.6, D 15.6, W 6.5)
“Drymaeusmeesi Breure, 1976”—Tillier 1980: 75, pl. 4 fig. 1, text-figs 60, 61.

##### Link.

https://molluscabase.org/aphia.php?p=taxdetails&id=1364218.

##### Material examined.

French Guiana • 1 dry specimen; Saül, Arbre à contreforts au niveau du belvédère de Saül, crête sud de Bœuf Mort (SAUL20); 3.62593°N, 53.21714°W; alt. 297 m; 16 Nov. 2018; OG, SS, ST, BF (PAG & MNHN) leg.; MNHN-IM-2012-21920 • 1 dry specimen; Saül (SAUL12); 3.61951°N, 53.20751°W; 08 Jul. 1999; TR, OG leg.; MNHN-IM-2012-21547 • 1 dry specimen; Saül (SAUL12); 3.61951°N, 53.20751°W; 08 Jul. 1999; TR, OG leg.; MNHN-IM-2013-21248.

##### Remarks.

The specimen illustrated by Tillier (1980: pl. 4 fig. 1) and Massemin et al. (2009: pl. 6A) identified as *Drymaeusmeesi* Breure, 1976 conchologically differs greatly from the holotype RMNH.MOL.55077 of *Drymaeusglaucostomusmeesi* Breure, 1976 (Breure 1976: pl. 1). Tillier (1980: 77) already stated that his species should be more related to *Drymaeusstrigatus* (G.B. Sowerby I (1838: fig. 95, original illustration) (Breure and Ablett 2014: fig. 30A–C, possible syntype NHMUK 20090168) rather than to *Drymaeusglaucostomus* and consequently elevated the taxon to species level. Indeed *Drymaeusstrigatus*, together with the synonymised *Bulimusmusivus* L. Pfeiffer, 1855 (Richardson 1995; Breure and Ablett 2014: fig. 29J–L, lectotype NHMUK 1975292), *Bulimussaccatus* L. Pfeiffer, 1855 (Breure and Ablett 2014: fig. 30D–F, lectotype NHMUK 1975207), as well as *Drymaeusschunkei* F. Haas (1949: 237, fig. 50b; Holotype FMNH 30040), all described from Peru, share the same global shape, chromatic pattern, and expanded aperture with the French Guianan species. However, they lack two characters that are shared between the French Guiana species and *Drymaeusarcuatostriatus*, i.e., the distinctive impressed radial white calcareous ribbing and the dark early whorls. Thus, we tentatively identify the French Guiana species previously known as “*Drymaeusmeesi*” sensu Tillier (1980) as *Drymaeusarcuatostriatus* L. Pfeiffer, 1855.

The species is to be considered within the Drymaeus (Drymaeus) expansus (L. Pfeiffer, 1848) species complex as defined by Breure and Borrero (2019), although the latter does not share the black early whorls.

**Figure 12.**
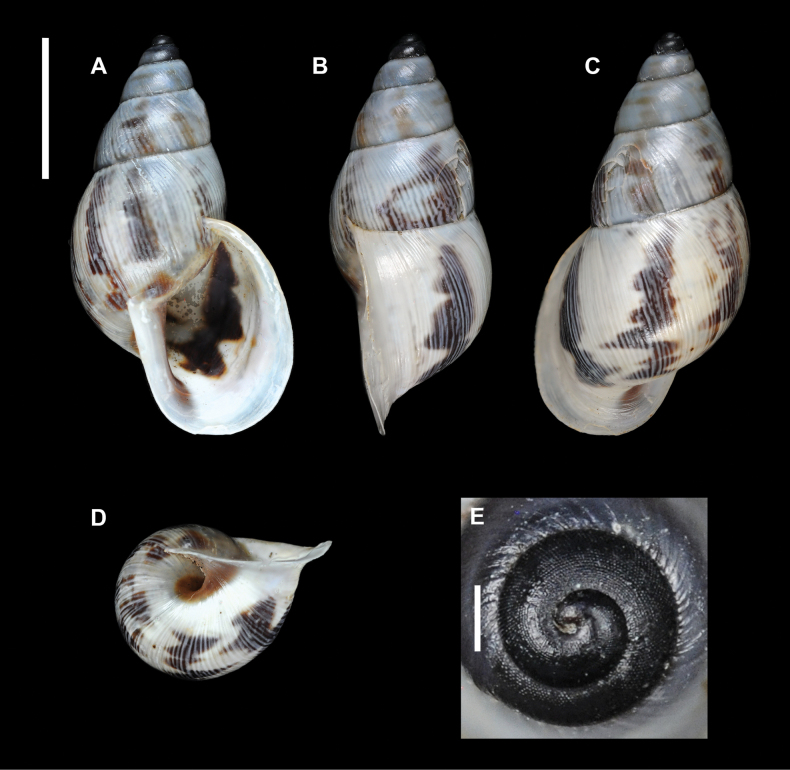
*Drymaeusarcuatostriatus*, Saül, MNHN-IM-2012-21547. Scale bars: 10 mm (**A–D**); 1 mm (**E**).

**Figure 13.**
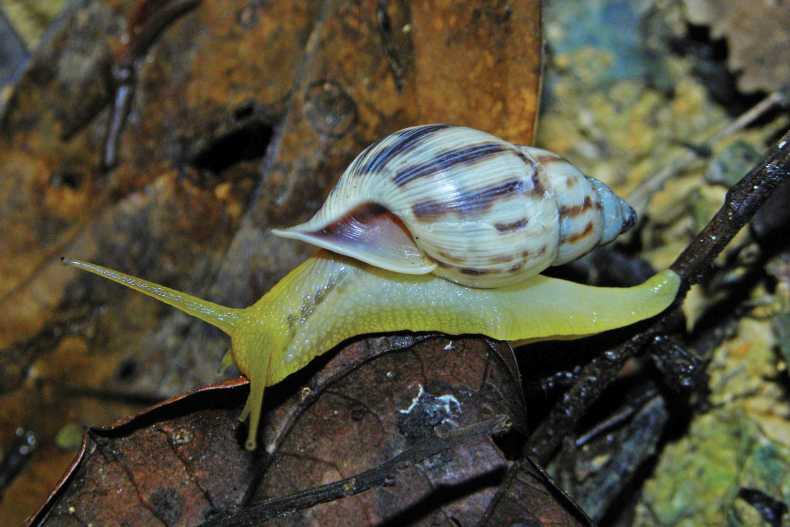
*Drymaeusarcuatostriatus*, Saül. Photograph by S. Sant (https://www.inaturalist.org/observations/74538163).

A living specimen (Fig. 13, https://www.inaturalist.org/observations/74538163) shows a yellow body, mostly on the sole and the head, whitish otherwise; longitudinal black lines extend from the bottom of the optical tentacle to the aperture. The optical tentacles are conical and elongated.

### ﻿Genus *Mesembrinus* Albers, 1850

#### ﻿ Mesembrinus lusorius

Taxon classificationAnimaliaArchitaenioglossaNeocyclotidae

(L. Pfeiffer, 1855)

0520B715-41B2-52E2-B6F9-A59BE7CC5141

Fig. 14

Bulimus lusorius L. Pfeiffer, 1855: 291; Breure (1979): 121, lectotype designation.
Drymaeus (Mesembrinus) lusorius —Breure and Eskens (1981): 77, pl. 7 fig. 12.
Mesembrinus lusorius —Simone (2006): 145, fig. 487.
Drymaeus surinamensis Vernhout, 1914: 13, pl. 1 fig. 3. syn. nov.
Mesembrinus surinamensis (Vernhout, 1914)—MolluscaBase, “new comb. herein, based on the ranking of Mesembrinus by Salvador et al. (2023)” [https://molluscabase.org/aphia.php?p=taxdetails&id=1713521]

##### Link.

https://molluscabase.org/aphia.php?p=taxdetails&id=1713485.

##### Material examined.

French Guiana • 1 dry specimen; Apatou; 1931–1932; BOUGE leg.; MNHN-IM-2023-4121 • 1 95% ethanol specimen; Régina, RN des Nouragues, carré F15 du km^2^ layonné (NB7); 4.08755°N, 52.68241°W; alt. 150 m; 03 Jun. 1999–29 Jun. 1999; TR, OG leg.; MNHN-IM-2013-87076 • 1 dry specimen; Régina, RN des Nouragues, carré N19 du km^2^ layonné (N11); 4.08083°N, 52.67617°W; alt. 90 m; 12 Nov. 1997; TR, OG leg.; forêt primaire, bord de pinotière; MNHN-IM-2023-4120 • 2 dry specimens; Saül, Mont Galbao face nord-est (SAUL66); 3.59995°N, 53.26697°W; alt. 382 m; 20 Feb. 2020; BF, AA, OG (PAG & MNHN) leg.; MNHN-IM-2018-14136 • 1 dry specimen; Saül, Camp Galbao (SAUL46); 3.6015°N, 53.27498°W; alt. 628 m; 26 Nov. 2018; OG, SS, ST, BF (PAG & MNHN) leg.; Bord de crique; MNHN-IM-2018-684 • 1 95% ethanol specimen; Saül, Camp Galbao (SAUL46); 3.6015°N, 53.27498°W; alt. 628 m; 26 Nov. 2018; OG, SS, ST, BF (PAG & MNHN) leg.; Bord de crique; MNHN-IM-2013-75878.

##### Remarks.

*Bulimuslusorius* L. Pfeiffer, 1855 was described from the “Banks of Amazon, Brazils” (Pfeiffer 1855). The lectotype NHMUK 1975543 illustrated in Breure and Ablett (2014: fig. 17A–C) and the specimen illustrated by Simone (2006: fig. 487) are morphologically similar to the syntype of *Drymaeussurinamensis* described by Vernhout (1914) (RMNH.MOL.335814) and with all specimens identified as this species from French Guiana where it is the commonest species of its genus s.l. Thus, we suggest that *Drymaeussurinamensis* Vernhout, 1914 is a junior synonym of *Bulimuslusorius* L. Pfeiffer, 1855 until further investigations, in particular molecular data, are carried out.

**Figure 14.**
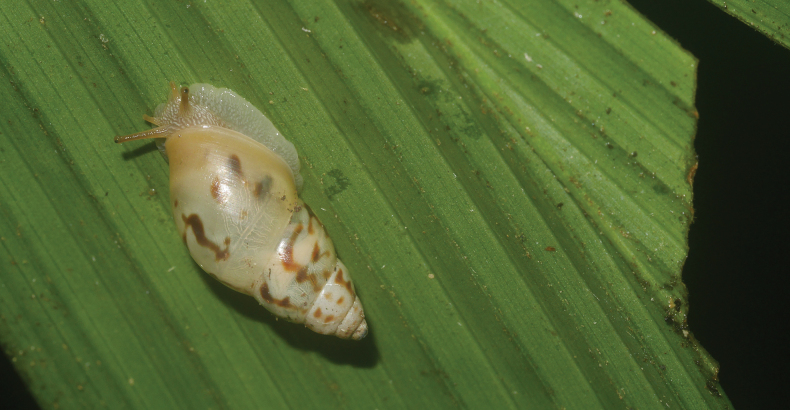
*Mesembrinuslusorius*, juvenile specimen on Mont Galbao, Saül, 630 m a.s.l., MNHN-IM-2013-75878.

Described as a subgenus of *Drymaeus*, *Mesembrinus* has been elevated to genus rank by Salvador et al. (2023).

Specimen A of Simone (2006: fig. 453, NMW.1955.158) as well as specimen RMNH.MOL.266069, both identified as *Drymaeusgermaini* (Ancey, 1892), might also belong to the species.

### ﻿Infraorder Pupilloidei

**Superfamily Pupilloidea W. Turton, 1831**

**Family Strobilopsidae Wenz, 1915**

**Genus *Strobilops* Pilsbry, 1893**

#### ﻿ Strobilops morsei

Taxon classificationAnimaliaArchitaenioglossaNeocyclotidae

(Dall, 1885)

8E696725-E5FB-5AE6-B0F6-B7C4871A5A3E

Figs 15 , 16

Strobila labyrinthica var. morsei Dall, 1885: 263.
Strobilops morsei —Pilsbry (1927–1935): 39, pl. 6 figs 4, 6 [type], pl. 6 fig. 5.
Strobila labyrinthica morsei —Baker (1925): 3; Altena (1975): 36.

##### Link.

https://molluscabase.org/aphia.php?p=taxdetails&id=1497894.

##### Material examined.

French Guiana • 1 95% ethanol specimen; Maripasoula, Massif du Mitaraka, Sommet en Cloche (mitaraka02); 2.22804°N, 54.467°W; alt. 599 m; 12 Mar. 2015; OG, BF leg.; inselberg avec bromeliacées; GenBank: PQ629108; Bold: DREAL1035-23; MNHN-IM-2013-77374 • 1 95% ethanol specimen; Maripasoula, Massif du Mitaraka, Sommet en Cloche (mitaraka34); 2.2316°N, 54.46109°W; alt. 339 m; 22 Mar. 2015; OG, BF leg.; bord de ruisseau avec rochers; GenBank: PQ629095; Bold: DREAL1036-23; MNHN-IM-2013-77375 • 3+1 dry specimens; same data as preceding; MNHN-IM-2012-21268, MNHN-IM-2012-21266 • 5+1 dry specimens; Maripasoula, Massif du Mitaraka, Sommet en Cloche (mitaraka36); 2.22847°N, 54.46721°W; alt. 586 m; 24 Mar. 2015; OG leg.; Forêt sommitale; MNHN-IM-2012-21267 and MNHN-IM-2012-21265.

**Figure 15.**
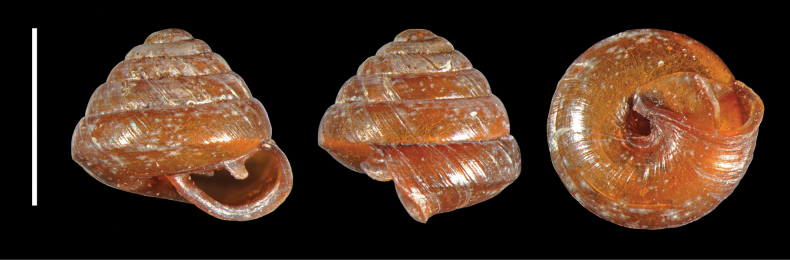
*Strobilopsmorsei* from Mitaraka, MNHN-IM-2012-21265. Scale bar: 2 mm.

##### Remarks.

This species was described from Venezuela (Dall 1885) and reported from Surinam as early as 1975 (Altena 1975). The studied material represents the first record of the family Strobilopsidae in French Guiana (Gargominy and Fontaine 2015).

**Figure 16.**
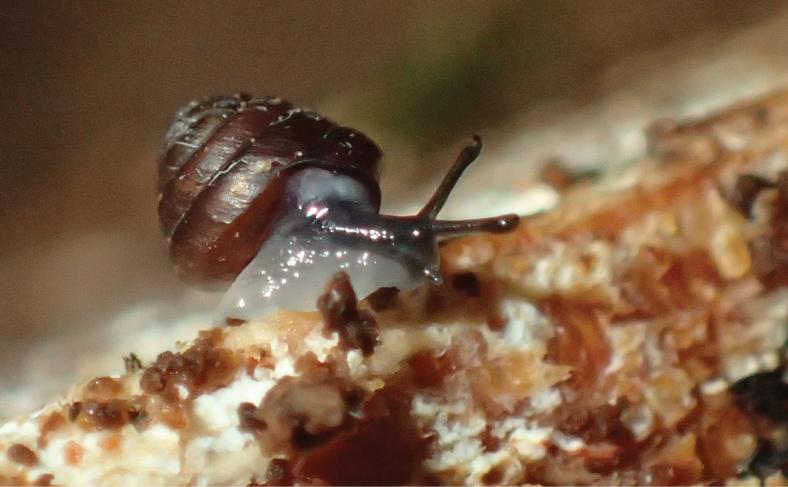
*Strobilopsmorsei* from Mitaraka, MNHN-IM-2012-21266.

In French Guiana, *Strobilopsmorsei* is only known from the Mitaraka Mountains between 340 and 600 m a.s.l.

The two COI sequences (same haplotype) are related to some *Strobilopslabyrinthicus* from Canada (*p*-distance 7.2–8.1%).

### ﻿Family Valloniidae Morse, 1864

**Genus *Pupisoma* Stoliczka, 1873**

#### ﻿ Pupisoma macneilli

Taxon classificationAnimaliaArchitaenioglossaNeocyclotidae

(G.H. Clapp, 1918)

4AEC5654-12E3-54E6-AF65-03AE9695D52A

Thysanophora macneilli G.H. Clapp, 1918: 74, pl. 8 fig. 1.
Pupisoma macneilli (G.H. Clapp, 1918)— Hausdorf (2007: 1497, figs 9, 10, 14).

##### Link.

http://molluscabase.org/aphia.php?p=taxdetails&id=1299519.

##### Material examined.

French Guiana • 25 dry specimens; Cayenne, Ilet la Mère (carré J14 de l’Institut Pasteur) (GUY34); 4.89102°N, 52.18392°W; alt. 10 m; 28 Nov. 1997; TR, OG leg.; forêt anciennement dégradée, pied d’un fromager; MNHN-IM-2018-14186 • 2 dry specimens; Régina, RN des Nouragues, carré J12 du km^2^ layonné (N3); 4.0883°N, 52.67675°W; alt. 80 m; 04 Nov. 1997; TR, OG leg.; forêt primaire, pied d’arbre à contrefort (code J-12-22); MNHN-IM-2018-14216 • 1 dry specimen; Régina, RN des Nouragues, carré E19 du km^2^ layonné (N9); 4.08487°N, 52.68361°W; alt. 110 m; 10 Nov. 1997; TR, OG leg.; forêt primaire, fond de talweg; MNHN-IM-2018-14197 • 1 dry specimen; Régina, RN des Nouragues, carré I14 du km^2^ layonné (N19); 4.08712°N, 52.67874°W; alt. 70 m; 19 Nov. 1997; TR, OG leg.; fond de vallée, pied d’arbre à contrefort (code I-14-187); MNHN-IM-2018-14199 • 1 dry specimen; Saül, Arbre à contreforts au niveau du belvédère de Saül, crête sud de Bœuf Mort (SAUL20); 3.62593°N, 53.21714°W; alt. 297 m; 16 Nov. 2018; OG, SS, ST, BF (PAG & MNHN) leg.; MNHN-IM-2012-21926 • 4 95% ethanol specimens; Saül, Sommet Mont Galbao (SAUL48); 3.60427°N, 53.28153°W; alt. 701 m; 28 Nov. 2018; OG, SS, ST, BF (PAG & MNHN) leg.; MNHN-IM-2013-75677 • 3 dry specimens; Saül, Bœuf mort (SAUL52); 3.63691°N, 53.21442°W; alt. 380 m; 15 Feb. 2020; BF, AA, OG (PAG & MNHN) leg.; Pied d’arbre à contrefort sous lianes et cambrouse à *Guadua*; MNHN-IM-2018-14154 • 9 dry specimens; Saül, Sommet Bœuf Mort (SAUL53); 3.63546°N, 53.21453°W; alt. 400 m; 15 Feb. 2020; BF, AA, OG (PAG & MNHN) leg.; Pied d’arbre à contrefort; MNHN-IM-2018-14128 • 9 dry specimens; Saül, Mont Galbao face nord-est (SAUL65); 3.59681°N, 53.26257°W; alt. 503 m; 20 Feb. 2020; BF, AA, OG (PAG & MNHN) leg.; Cambrouse et lianes en forêt; MNHN-IM-2018-14052 • 1 dry specimen; Saül, Mont Galbao face nord-est (SAUL68); 3.60018°N, 53.25877°W; alt. 251 m; 21 Feb. 2020; BF, AA, OG (PAG & MNHN) leg.; Bord de cambrouse, sous lianes; MNHN-IM-2018-14033 • 1 95% ethanol specimens; Mana, roche bénitier, 200m avant abri arca (sommet inselberg) (TRI12); 4.62154°N, 53.40515°W; alt. 429 m; 12 Apr. 2019; BF, ST (MNHN/ONF) leg.; Forêt en pied de falaise granitique; MNHN-IM-2013-75760 • 1 dry specimen; Mana, Layon A 1350 m du camp Aya (TRI15); 4.59933°N, 53.42685°W; alt. 129 m; 13 Apr. 2019; BF, ST (MNHN/ONF) leg.; Forêt de plateau; MNHN-IM-2013-75761.

##### Remarks.

We revised all *Pupisoma* species we collected in French Guiana according to Hausdorf (2007), which allowed us to recognize *Pupisomamacneilli* as a new record for French Guiana, in addition to the previously known *Pupisomadioscoricola* (C.B. Adams, 1845).

### ﻿Infraorder Succineoidei

**Superfamily Testacelloidea Gray, 1840**

**Family Spiraxidae H.B. Baker, 1939**

**Subfamily Spiraxinae H.B. Baker, 1939**

**Genus *Pseudosubulina* Strebel & Pfeffer, 1882**

#### ﻿ Pseudosubulina santi

Taxon classificationAnimaliaArchitaenioglossaNeocyclotidae

Gargominy sp. nov.

3B188F7E-6BB3-5D51-B41B-32702C3A5A4C

https://zoobank.org/7EE76DD9-3553-4A00-8BFD-0C6B808A447B

Figs 17 , 18

##### Type locality.

French Guiana, in the vicinity of Saül village, southern foothill of Bœuf Mort mountain.

##### Type material.

***Holotype*.** French Guiana • 1 dry specimen; Saül, Arbre à contreforts au niveau du belvédère de Saül, crête sud de Bœuf Mort (SAUL20); 3.62593°N, 53.21714°W; alt. 297 m; 16 Nov. 2018; OG, SS, ST, BF (PAG & MNHN) leg.; MNHN-IM-2012-21923. ***Paratypes*** (8). French Guiana • 2 95% ethanol specimens; Saül, Bananeraie, Crique gros fossé (SAUL51); 3.6288°N, 53.21246°W; alt. 201 m; 14 Feb. 2020; BF, AA, OG (PAG & MNHN) leg.; Cambrouse à *Guadua*; MNHN-IM-2013-76444; MNHN-IM-2013-76882 (both tentatively barcoded without success) • 6 dry specimens; same data as preceding; MNHN-IM-2018-14018.

##### Other material examined.

French Guiana • 1 95% ethanol specimens; Saül, Bœuf Mort (SAUL54); 3.63966°N, 53.21413°W; alt. 350 m; 15 Feb. 2020; BF, AA, OG (PAG & MNHN) leg.; MNHN-IM-2013-76486 (tentatively barcoded without success) • 1 95% ethanol specimen; Saül, Crête au sud de crique cochon (SAUL58); 3.61427°N, 53.19857°W; alt. 127 m; 16 Feb. 2020; BF, AA, OG (PAG & MNHN) leg.; Pied d’arbre à contrefort et écorces mortes au sol; MNHN-IM-2013-76456 (tentatively barcoded without success) • 1 95% ethanol specimens; Saül, Abattis en arrière du village (SAUL60); 3.62609°N, 53.21105°W; alt. 162 m; 17 Feb. 2020; BF, AA, OG (PAG & MNHN) leg.; Forêt bordant le village; MNHN-IM-2013-76488 (tentatively barcoded without success) • 2 dry specimens; same data as preceding; MNHN-IM-2018-14165 • 2 dry specimens; Saül, Mont Galbao (SAUL47); 3.60183°N, 53.27239°W; alt. 650 m; 27 Nov. 2018; OG, SS, ST, BF (PAG & MNHN) leg.; DZ brulée et cambrouse à l’est; MNHN-IM-2012-21982 • 1 dry specimen; Saül, Cascades du Mont Galbao, face nord-est (SAUL64); 3.60376°N, 53.26121°W; alt. 320 m; 19 Feb. 2020; BF, AA, OG (PAG & MNHN) leg.; Bordure de cambrouse; MNHN-IM-2018-14063 • 1 dry specimen; Saül, Mont Galbao face nord-est (SAUL65); 3.59681°N, 53.26257°W; alt. 503 m; 20 Feb. 2020; BF, AA, OG (PAG & MNHN) leg.; Cambrouse et lianes en forêt; MNHN-IM-2018-14042.

**Figure 17.**
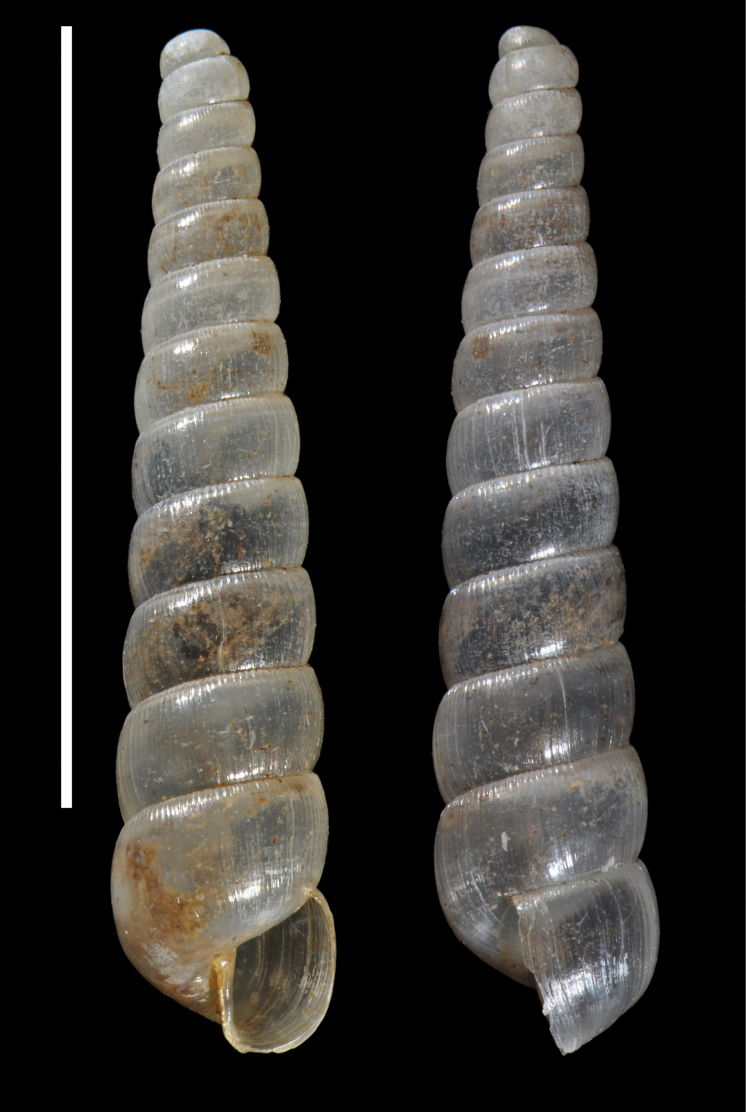
*Pseudosubulinasanti* sp. nov. holotype MNHN-IM-2012-21923. Scale bar: 10 mm.

##### Diagnosis.

A *Pseudosubulina* of normal size characterised by its absence of sculpture on the shell where only growth lines are visible.

##### Description.

Holotype. Shell of normal size for the genus (height 13.1 mm, major diameter 2.9 mm), dextral, slender, turreted with straight outline, glossy, rather thin, semi-translucent, white. Whorls 13, rounded, slightly flattened at the periphery, with a rather deep straight crenulated suture all the way to the aperture. Protoconch smooth, protoconch/teleoconch transition indistinct. Surface of teleoconch with very fine and numerous growth lines. Aperture elongated, orthocline, with slightly more than 90° columellar-basal angle, cut by penultimate whorl. Peristome simple, with sharp margin, not reflected except at columellar edge; reflection of columellar edge more developed in its upper part. Umbilicus tiny, partially covered by columellar edge.

##### Etymology.

The species is named after Sébastien Sant, a good friend and experienced botanist and naturalist who helped us so much during our field trip in Saül.

##### Distribution.

This species is only known from French Guiana in the vicinity of Saül village, including the southern foothill of Mont Galbao.

##### Habitat.

Primary forest, under leaf litter on granitic or lateritic soil.

##### Remarks.

**Figure 18.**
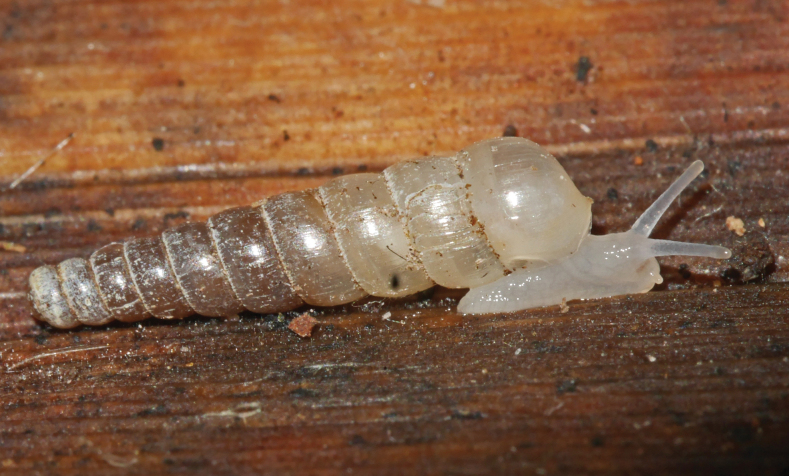
*Pseudosubulinasanti* sp. nov. from Saül, paratype MNHN-IM-2013-76882.

Paratype (MNHN-IM-2013-76882) has the body white; first ~ 7 whorls darker because of dark hepatopancreas; tentacles elongated, white, a little darker at the tip; eyes not distinctly visible (Fig. 18).

Four specimens were barcoded without success.

*Pseudosubulinasanti* sp. nov. is the third species of the genus recorded from French Guiana. Although it is known only from Saül area, it is not rare there: it is recorded in the vicinity of the village as well as on Mont Galbao, i.e., at altitudes between 125 and 650 m a.s.l.

*Pseudosubulinasanti* sp. nov. is found in syntopy with *Pseudosubulinatheoripkeni* Gargominy & Muratov, 2012.

#### ﻿ Pseudosubulina theoripkeni

Taxon classificationAnimaliaArchitaenioglossaNeocyclotidae

Gargominy & Muratov, 2012

777C3183-FB30-539A-8F13-6AB2310EA047

Fig. 19

Pseudosubulina theoripkeni Gargominy & Muratov, 2012: 786, figs 3, 4.

##### Link.

https://molluscabase.org/aphia.php?p=taxdetails&id=1477357.

##### Type locality.

French Guiana, Régina, Réserve naturelle des Nouragues, Montagnes Balenfois, field station.

##### Type material.

***Holotype*.** French Guiana • Régina, RN des Nouragues, carré G17 du km^2^ layonné (N1); 4.08524°N, 52.68179°W; alt. 75 m; 03 Nov. 1997; TR, OG leg.; forêt primaire, bord de ruisseau, bloc granitique; MNHN-IM-2000-25068. ***Paratypes*** (8). French Guiana 1 95% ethanol specimen and 1 dry specimen; same data as the holotype; MNHN-IM-2000-25069 and MNHN-IM-2000-25070 respectively • 5 dry specimens; Régina, RN des Nouragues, carré M12 du km^2^ layonné (N7); 4.08699°N, 52.67381°W; alt. 140 m; 08 Nov. 1997; TR, OG leg.; forêt primaire, grand plateau; MNHN-IM-2000-25071 • 1 dry specimen; Régina, RN des Nouragues, carré N15 du km^2^ layonné (N14); 4.08372°N, 52.67503°W; alt. 155 m; 15 Nov. 1997; TR, OG leg.; forêt primaire, pied d’arbre à contrefort (code N-15-138); MNHN-IM-2000-25072.

##### Other material examined.

French Guiana • 1 dry specimen; Roura, Route de Cacao (Boulanger), 5.3 km après embranchement N2, 700 m après la scierie (COT13); 4.56548°N, 52.41922°W; alt. 100 m; 23 Oct. 1997; TR, OG leg.; Une parcelle brûlée et forêt adjacente; MNHN-IM-2018-14056 • 1 dry specimen; Régina, RN des Nouragues, carré L14 du km^2^ layonné (N2); 4.08564°N, 52.67651°W; alt. 110 m; 04 Nov. 1997; TR, OG leg.; forêt primaire, forêt de lianes; MNHN-IM-2018-14180 • 3 dry specimens; Régina, RN des Nouragues, carré M12 du km^2^ layonné (N7); 4.08699°N, 52.67381°W; alt. 140 m; 08 Nov. 1997; TR, OG leg.; forêt primaire, grand plateau; MNHN-IM-2018-14057 • 1 dry specimen; Régina, RN des Nouragues, carré E19 du km^2^ layonné (N9); 4.08487°N, 52.68361°W; alt. 110 m; 10 Nov. 1997; TR, OG leg.; forêt primaire, fond de talweg; MNHN-IM-2018-14182 • 7 dry specimens; Régina, RN des Nouragues, carré N10 du km^2^ layonné (N10); 4.08845°N, 52.67269°W; alt. 140 m; 11 Nov. 1997; TR, OG leg.; forêt primaire, chablis; MNHN-IM-2018-14055 • 2 dry specimens; Régina, RN des Nouragues, carré N15 du km^2^ layonné (N14); 4.08372°N, 52.67503°W; alt. 155 m; 15 Nov. 1997; TR, OG leg.; forêt primaire, pied d’arbre à contrefort (code N-15-138); MNHN-IM-2018-14181 • 1 dry specimen; Régina, RN des Nouragues, carré N13 du km^2^ layonné (NB5); 4.08603°N, 52.67353°W; alt. 170 m; 03 Jun. 1999–29 Jun. 1999; TR, OG leg.; MNHN-IM-2018-14295 • 1 dry specimen; Maripasoula, Mont Itoupé (Itoupé600); 3.02314°N, 53.09533°W; alt. 600 m; 06 Jan. 2016–17 Jan. 2016; Thibaud Decaëns, Sébastien Cally leg.; MNHN-IM-2012-21480 • 1 95% ethanol specimen; Maripasoula, Mont Itoupé (Itoupé600); 3.02314°N, 53.09533°W; alt. 600 m; 06 Jan. 2016–17 Jan. 2016; Thibaud Decaëns, Sébastien Cally leg.; MNHN-IM-2012-21482 • 2 95% ethanol specimens; Saül, Arbre à contreforts au niveau du belvédère de Saül (SAUL20); 3.62593°N, 53.21714°W; alt. 297 m; 16 Nov. 2018; OG, SS, ST, BF (PAG & MNHN) leg.; MNHN-IM-2013-75869; MNHN-IM-2013-75870 • 5 95% ethanol specimens; same data as preceding; MNHN-IM-2013-75597 • 14 dry specimens; same data as preceding; MNHN-IM-2012-21922 • 1 95% ethanol specimen; Saül, Versant nord de Bœuf Mort, le long du sentier de Grand Bœuf Mort (SAUL44); 3.64098°N, 53.21863°W; alt. 300 m; 24 Nov. 2018; OG, SS, ST, BF (PAG & MNHN) leg.; Pied d’arbre à contreforts; MNHN-IM-2013-75604 • 8 dry specimens; Saül, Mont Galbao (SAUL47); 3.60183°N, 53.27239°W; alt. 650 m; 27 Nov. 2018; OG, SS, ST, BF (PAG & MNHN) leg.; DZ brulée et cambrouse à l’est; MNHN-IM-2012-21981 • 2 95% ethanol specimens; Saül, Sommet Bœuf Mort (SAUL53); 3.63546°N, 53.21453°W; alt. 400 m; 15 Feb. 2020; BF, AA, OG (PAG & MNHN) leg.; Pied d’arbre à contrefort; MNHN-IM-2013-77069 and MNHN-IM-2013-77070 • 5 dry specimens; same data as preceding; MNHN-IM-2018-14125 • 2 95% ethanol specimens; Saül, Chemin des Monts la Fumée (SAUL63); 3.63119°N, 53.20586°W; alt. 177 m; 18 Feb. 2020; BF, AA, OG (PAG & MNHN) leg.; Pied d’arbre à contrefort; MNHN-IM-2013-76420 • 1 95% ethanol specimen; Saül, Mont Galbao face nord-est (SAUL65); 3.59681°N, 53.26257°W; alt. 503 m; 20 Feb. 2020; BF, AA, OG (PAG & MNHN) leg.; Cambrouse et lianes en forêt; MNHN-IM-2013-76401 • 2 dry specimens; Saül, Mont Galbao face nord-est (SAUL66); 3.59995°N, 53.26697°W; alt. 382 m; 20 Feb. 2020; BF, AA, OG (PAG & MNHN) leg.; MNHN-IM-2018-14141 • 1 dry specimen; Saül, Piste Limonade (SAUL71); 3.58813°N, 53.21204°W; alt. 170 m; 24 Feb. 2020; BF, AA, OG (PAG & MNHN) leg.; Bord de cambrouse; MNHN-IM-2018-14172 • 1 dry specimen; Mana (TRI05); 4.59397°N, 53.4146°W; alt. 94 m; 09 Apr. 2019; BF, ST (MNHN/ONF) leg.; MNHN-IM-2014-7865 • 1 dry specimen; Mana, Abri Arca (sommet inselberg) (TRI10); 4.62181°N, 53.40359°W; alt. 429 m; 11 Apr. 2019; BF, ST (MNHN/ONF) leg.; grotte; MNHN-IM-2014-7866.

##### Description of external body.

**Figure 19.**
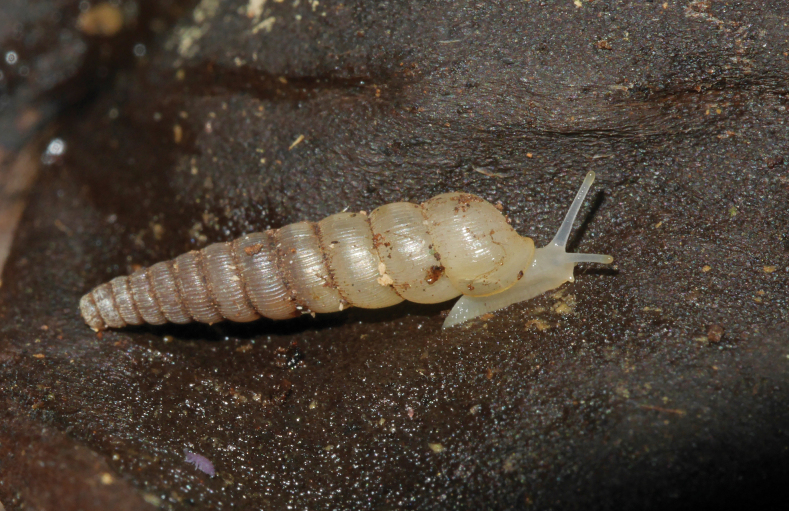
*Pseudosubulinatheoripkeni* from Saül, southern foothills of Bœuf Mort, MNHN-IM-2013-75869.

Body white, first ~ 8 whorls darker because of dark hepatopancreas; tentacles almost cylindrical but a little larger at the base, with thickening, pale brownish tip; eyes not distinctly visible (Fig. 19).

##### Distribution.

Roura, Saül (including Mont Galbao), Itoupé, and Montagnes de la Trinité are new records for this species previously only known from its type locality in Nouragues (Gargominy and Muratov 2012), suggesting a much broader distribution probably extending outside French Guiana.

##### Remarks.

The first living specimens ever found are reported here; they were barcoded without success.

### ﻿Suborder Scolodontina

**Superfamily Scolodontoidea H.B. Baker, 1925**

**Family Scolodontidae H.B. Baker, 1925**

**Genus *Happia* Bourguignat, 1890**

#### ﻿ Happia decaensi

Taxon classificationAnimaliaArchitaenioglossaNeocyclotidae

Gargominy sp. nov.

811D39DD-27F0-50AE-9D54-28B20EE9CAC2

https://zoobank.org/DA2519E0-8142-4AD2-8081-572CDC0C4833

Fig. 20

##### Type locality.

French Guiana, Camopi, Mont Itoupé.

##### Type material.

***Holotype*.** French Guiana • 1 95% ethanol specimen (shell separated); Camopi, Mont Itoupé; 3.02696°N, 53.07902°W; alt. 800 m; 06 Jan. 2016–17 Jan. 2016; Thibaud Decaëns, Sébastien Cally leg.; MNHN-IM-2013-75995. ***Paratypes*** (7). French Guiana • 4 dry specimens; Régina, RN des Nouragues, carré J12 du km^2^ layonné (N3); 4.0883°N, 52.67675°W; alt. 80 m; 04 Nov. 1997; TR, OG leg.; forêt primaire, pied d’arbre à contrefort (code J-12-22); MNHN-IM-2018-14023, MNHN-IM-2018-14429, MNHN-IM-2018-14430, MNHN-IM-2018-14431 • 1 dry specimen; Régina, RN des Nouragues, carré G12 du km^2^ layonné (N16); 4.09011°N, 52.67922°W; alt. 80 m; 16 Nov. 1997; TR, OG leg.; forêt primaire, bord de Crique Couac (rive droite); MNHN-IM-2018-14024 • 2 dry specimens; Régina, RN des Nouragues, carré I17 du km^2^ layonné (N17); 4.08535°N, 52.67996°W; alt. 73 m; 17 Nov. 1997; TR, OG leg.; confluence crique Nouragues et crique Moteur, forêt primaire, accumulation de bois pourri; MNHN-IM-2018-14022, MNHN-IM-2018-14428.

##### Diagnosis.

A large *Happia* species with relatively high whorls, strong indentation on the upper sutural margin, largely umbilicated.

##### Description.

Holotype. Shell small (height 2.8 mm, greater diameter 5.6 mm), dextral, totally depressed, thin; colour pale corneous; whorls 3.8, inflated, rounded, slightly flattened on the upper part, overlapping the preceding, separated by a marked suture; spire planispiral, coiling rapidly increasing. Protoconch 1.7 whorl, with ~ 10 delicate spiral threads hardly visible due to periostracal erosion, also visible from adapical view; protoconch/teleoconch transition distinct, particularly from adapical view; teleoconch smooth (but see remarks below), with sigmoid, prominent, closely spaced growth wrinkles with irregular levels of shell calcification underneath, radial on umbilical part of whorls, strongly rounded backwards near upper suture. Body whorl rounded, strongly flattened above periphery up to a small bulge above the suture. Aperture prosocline, strongly sigmoid with an acute and deep (1 mm) incision in parietal angle, basally circular. Peristome simple, sharp. Umbilicus very large, almost half of the greater diameter, conical with flat protoconch entirely visible.

##### Etymology.

The species is named after Thibaud Decaëns who provided the holotype and only living specimen, a good friend and experienced earthworm expert.

##### Distribution.

This species is known from French Guiana only, Nouragues and Itoupé.

##### Habitat.

Leaf litter of tropical rain forest, from 80 (Nouragues) to 800 m (Itoupé) a.s.l.

##### Remarks.

The holotype was barcoded, without success.

**Figure 20.**
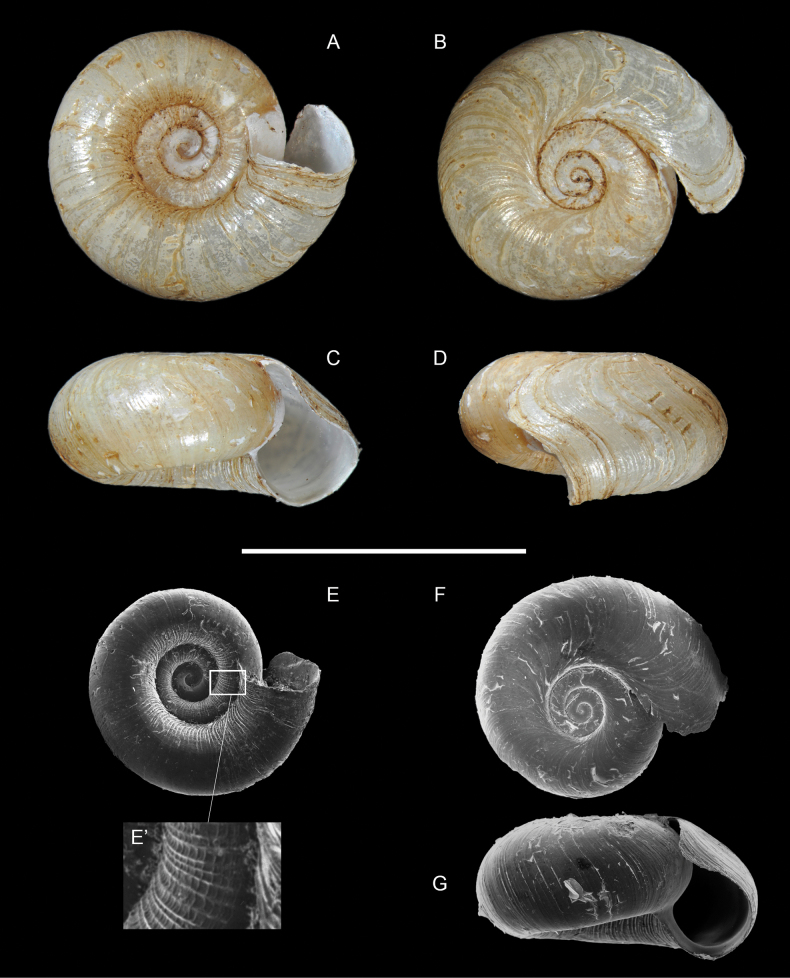
*Happiadecaensi* sp. nov.: **A–D** holotype MNHN-IM-2013-75995**E** paratype MNHN-IM-2018-14429 **F** paratype MNHN-IM-2018-14430 **G** paratype MNHN-IM-2018-14431. Scale bar: 5 mm.

The paratype MNHN-IM-2018-14022 is a juvenile (2.7 whorls) and distinctly shows the spiral rows of minute papillae on the protoconch; this spiral micro-sculpture is also visible on the complete teleoconch which argues for its attribution to genus *Happia* (Baker 1925: pl. 8 fig. 35; Roosen and Breure 2024). Paratype MNHN-IM-2018-14429 (3.7 whorls) also shows this spiral micro-sculpture on the umbilical part of the teleoconch whorls, thus appearing reticulate when crossed with the marked growth lines (Fig. 20E’).

Referring to Roosen and Breure (2024), the new species is the largest of the genus. The type species *Happiaammonoceras* (L. Pfeiffer, 1855) (syntype NHMUK 20210342) from Colombia is smaller, flatter, the body whorl more inflated. *Drepanostomellapinchoti* Pilsbry, 1930 (holotype ANSP152648) described from Panama and recorded from Venezuela (Thompson 1957) is smaller with the umbilicus only 1/3 of the diameter. When compared to a juvenile of the new species (of the same size), it is more discoid and flatter.

This new species represents the first record of the genus *Happia* in French Guiana.

### ﻿Order Systellommatophora Pilsbry, 1948

**Superfamily Veronicelloidea J. E. Gray, 1840**

**Family Veronicellidae J. E. Gray, 1840**

**Genus *Diplosolenodes* Thomé, 1975**

#### ﻿ Diplosolenodes occidentalis

Taxon classificationAnimaliaArchitaenioglossaNeocyclotidae

(Guilding, 1825)

9859A8F5-C529-5DCE-B3E9-6B53DCF725A9

Fig. 21

Onchidium occidentale Guilding, 1825: 323.

##### Link.

https://molluscabase.org/aphia.php?p=taxdetails&id=1064168.

##### Material examined.

French Guiana • 2 95% ethanol specimens; Saül, Dans le village sur la piste (SAUL22); 3.62386°N, 53.21075°W; alt. 173 m; 16 Nov. 2018; OG, SS, ST, BF (PAG & MNHN) leg.; GenBank: PQ629109; Bold: DREAL580-23; MNHN-IM-2013-75891; GenBank: PQ629110; Bold: DREAL579-23; MNHN-IM-2013-75892.

##### Remarks.

These two specimens were collected at night on a track within the village of Saül and nowhere outside the village, which argues for a human introduction rather than a natural occurrence. Identification is based on the COI sequences. The same haplotype recovered from two specimens matches with a Suriname specimen (*p*-distance 99.83%, GenBank KM489511; Gomes, S.R., Barr, N. and Robinson, D., 2015, unpubl.).

**Figure 21.**
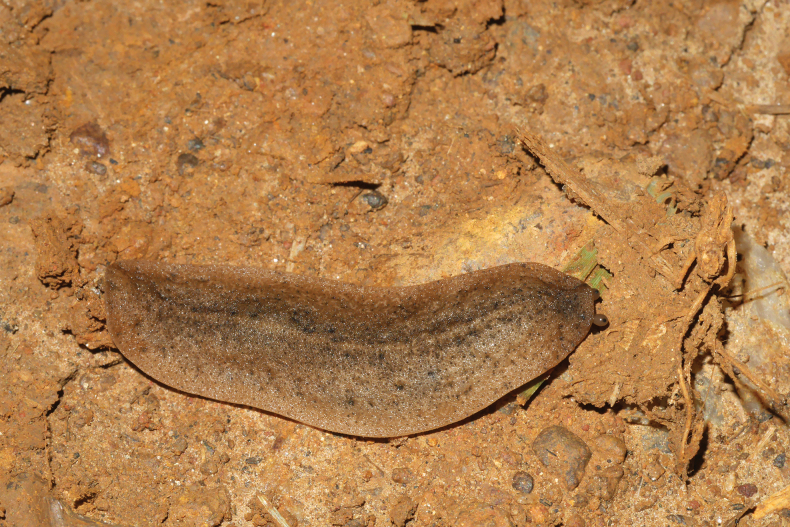
*Diplosolenodesoccidentalis* from Saül village, MNHN-IM-2013-75891.

## ﻿Discussion

The French Guiana land gastropod fauna (81 species) is surprisingly not diverse when compared to other groups from the same area: 15,100 species of insects (Brûlé and Touroult 2014) revised to 19,151 ten years later (TAXREF 2024), 476 species of spiders (Vedel et al. 2013; TAXREF 2024), and 134 species of amphibians (Lescure et al. 2022). This paper exemplifies the fact that this fauna is very poorly known, with each single collecting trip revealing new records, species new to science, or even new family records for the territory.

### ﻿Total number of extant species

There are currently 81 known land gastropod species in French Guiana, including 13 introduced or cryptogenic ones (this paper, TAXREF 2024). Among these 68 native species, eight (11.8%) have been described since 2010, a pattern reminiscent of that observed in Brazil, with 7% of the ~ 700 known species having been described after 2005 (Salvador 2019). Perhaps fuelled by an awareness of this unexpected richness, and by the recent use of leaf-litter sieving to reveal minute and rare species, there has been a renewed interest for the malacofauna of this region since the beginning of the 21^st^ century. However, even large species may still be discovered, as shown by the example of *Pseudosubulinasanti*, a relatively large (1 cm) species new to science, discovered with the naked eye in a banana plantation just outside the village of Saül, i.e., in an easily reachable area. These knowledge gaps are also shown by another example from the same area, the discovery of a previously unmentioned large introduced species, *Diplosolenodesoccidentalis*. Since the 1997 collecting trips in Nouragues, each collection campaign has brought its share of discoveries, including species new to science (e.g., this paper; Gargominy and Muratov 2012), and there is no doubt that any new collecting trip by experienced collectors would reveal new records and new species. Another indication of our lack of knowledge is the fact that the MNHN collections harbour several old specimens of unrecorded species, in particular 27 specimens of an Urocoptidae (possibly genus *Macroceramus*) labelled “Guyane franc, M Etienne, 1877” (MNHN-IM-2018-945) which needs confirmation. However, the most important reservoir of new species definitely consists of a micro-fauna of Scolodontidae, living in forest litter and observed as early as 1998 (Gargominy and Ripken 1998); these species are a few millimetres in size, have translucent shells and yellowish, pinkish, or purple bodies. In this context, it would be very hazardous to assess the expected total number of land snail species in French Guiana.

### ﻿Under-surveyed areas

**Figure 22.**
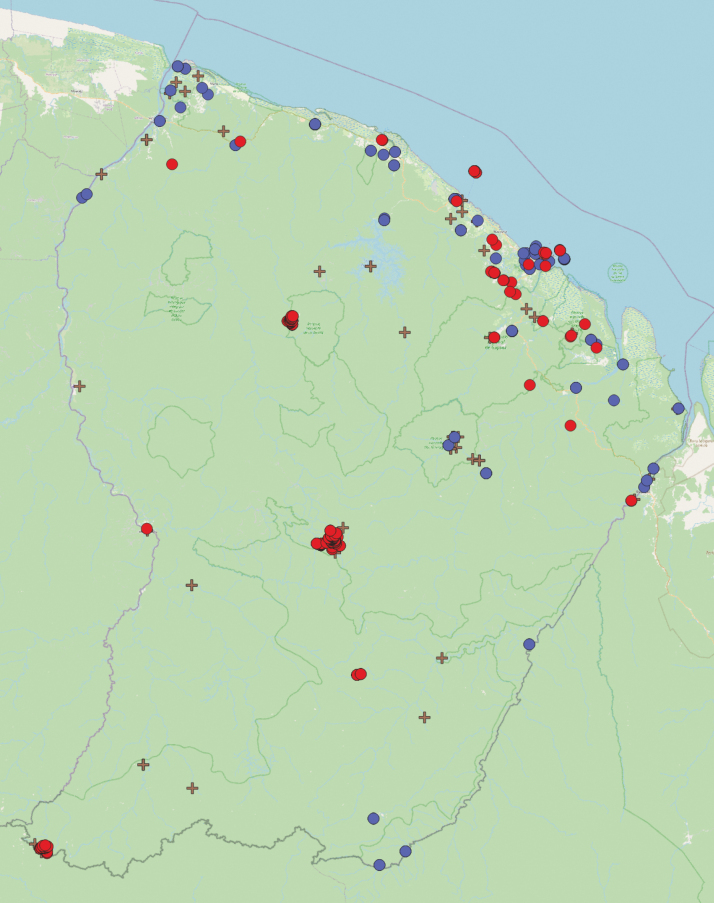
Map of sample localities for land snails in French Guiana: pre-2000 samples (blue dots), post-1999 samples (red dots), and opportunistic large (mostly single) species samples (plus symbol; *Angustipescarceralis*, *Bulimuluseyriesii*, *Euglandinastriata*, *Labyrinthus* spp., *Lissachatinaimmaculata*, *Megalobulimusoblongus*, *Neocyclotus* spp., Orthalicidae, *Solaropsisundata*).

The logistical difficulties to reach the forested interior of French Guiana, together with the low abundance of most species, account for the fact that apart from the coastal zone, French Guiana is very poorly known. Fig. 22 shows that the vast majority of the French Guianan territory has never been visited by malacologists. This is especially striking when localities of large, conspicuous species such as *Sultanasultana* or *Solaropsisundata*, which are often collected or photographed by non-malacologist naturalists, are removed from the map: outside the coastal zone, the only places where numerous small species have been collected are Nouragues, Saül, Trinité, and Mitaraka, i.e., places where experienced malacologists have made extensive collections with litter sieving.

### ﻿Range of known species

Some recently described (*Cyclopedusanselini*, *Happiadecaensi*, *Pseudosubulinatheoripkeni*) or recorded species (*Lyroconusplagioptycha*, *Pupisomamacneilli*) appear to be potentially widespread in French Guiana, suggesting they have been overlooked by previous collectors. Recent collecting events have added four new families to French Guiana (this paper; Gargominy and Muratov 2012).

Conversely, many species are only known from their type localities, such as *Adelopomaquasimodo*, *Lilloiconchagalbao*, and *Pseudosubulinasanti* only known around Saül, or *Strobilopsmorsei* which is only known from Mitaraka. However, the vast majority of French Guiana has never been searched for land snails, it is therefore premature to draw any conclusions about the real range of these species.

### ﻿Genetics

Whenever possible, we have endeavoured to collect live specimens and extract DNA to obtain COI sequences (total of 126 specimens belonging to ~ 29 species). However, apart from introduced species (Veronicellidae, Subulininae), these sequences could not be analysed using phylogenetic trees, since there are very few available published genetic data for South American species. Thus, supplementary field work targeting live specimens for genetic material and massive sequencing is needed to complete our knowledge.

### ﻿Shelf life and turbo-taxonomy

The situation is similar for another soil invertebrate taxon, earthworms (Order Crassiclitellata), of which 42 described species are known from French Guiana (TAXREF 2024), including 18 described in 2024 (Decaëns et al. 2024). As with the terrestrial molluscs, a large proportion of the diversity of earthworms is still unknown, since a recent study using DNA barcoding has revealed an unsuspected diversity, with 119 putative species (Maggia et al. 2021).

All described species in this paper are mainly based on material collected in Saül, a shelf life of 6 years, with the exception of *Happiadecaensi* collected as early as 1997, a shelf life of 27 years. This is less than the 21 years of shelf life between discovery and description of 600 species randomly taken from among the 16,994 species described in 2007, fungi, plants, and animals together (Fontaine et al. 2012a). However, our collections have revealed a great diversity of Scolodontidae, with probably at least fifteen species that are new to science. We may have to wait a few years before they are described, since the taxonomic workforce is sparse, and relies heavily on non-professional taxonomists (none of the authors of the present paper is a professional taxonomist sensu Fontaine et al. 2012b). In conclusion, if we are to reduce the length of the shelf life, integrative and turbo-taxonomy is needed, particularly for diversified groups such as scolodontids.

## Supplementary Material

XML Treatment for Cyclopedus anselini

XML Treatment for Adelopoma quasimodo

XML Treatment for Lilloiconcha galbao

XML Treatment for Lyroconus plagioptycha

XML Treatment for Protoglyptus bernicolae

XML Treatment for Drymaeus arcuatostriatus

XML Treatment for Mesembrinus lusorius

XML Treatment for Strobilops morsei

XML Treatment for Pupisoma macneilli

XML Treatment for Pseudosubulina santi

XML Treatment for Pseudosubulina theoripkeni

XML Treatment for Happia decaensi

XML Treatment for Diplosolenodes occidentalis

## ﻿Additional information

### Author ORCIDs

Olivier Gargominy https://orcid.org/0000-0001-7807-944X

Benoît Fontaine https://orcid.org/0000-0002-1017-5643

Sandrine Tercerie https://orcid.org/0000-0002-4341-062X

Dario Zuccon https://orcid.org/0000-0001-8236-5924

### Data availability

All of the data that support the findings of this study are available in the main text.
